# Scrutinized the inherent spin half-metallicity and thermoelectric response of *f*-electron-based RbMO_3_ (M = Np, Pu) perovskites: a computational assessment

**DOI:** 10.1038/s41598-022-22633-y

**Published:** 2022-11-14

**Authors:** Mudasir Younis Sofi, Dinesh C. Gupta

**Affiliations:** 1 Department of Physics, Jamia Millia Islamia, New Delhi, 110025 India; 2 Condensed Matter Theory Group, School of Studies in Physics, Jiwaji University, Gwalior, 474011 India

**Keywords:** Materials science, Condensed-matter physics

## Abstract

In the hunt for novel materials, we present self-consistent ab initio simulations of the structural stability, electronic profile, and transport properties of *f*-electron-based RbMO_3_ (M = Np, Pu) perovskites within the context of density functional theory. The structural stability and thermodynamic concerns are fixed by relaxing the crystal structure and computing the energy of formation, respectively. Furthermore, the decisive physical features of given materials have been outlined using the optimised lattice constant retrieved from structural optimizations. The ground state magnetic phase stability is ascertained by minimizing Birch Murnaghan's equation of state in distinct magnetic phases, upholding the ferromagnetic phase (FM) as the ground state magnetic phase, which is further backed by positive Curie Wiess constant values. To specify the electronic structure, a mix of the two approximations GGA and GGA + mBJ has been executed, both of which assert the half-metallic character, culminating in 100% spin polarisation at the Fermi level. The study of the magnetic moment and Curie temperature of each material has further been assessed in the present study. Apart from half-metallicity, the thermoelectric response of the present materials is quantified by exploring the chemical potential dependency of several transport parameters like Seebeck coefficient, electrical and thermal conductivity, power factor, etc. Moreover, the thermoelectric competence has been tested using a zT calculation, adapting values of 1.01 and 0.987 at 300 K for RbNpO_3_ and RbPuO_3_, respectively. The high electronic zT at encompassing temperatures uncovers the significant utility of these materials in both low-and high-temperature thermoelectric device applications. In essence, the comprehensive survey of these alloys could certainly open up their possibilities in spintronics, thermoelectric, and solid-state (RTG) device applications.

## Introduction

The quest for novel materials with remarkable properties to comply with the requirements of current and forthcoming technologies is attaining new heights. Besides analytical and experimental approaches, DFT-based simulations have emerged as an efficient tool for tracking down and analysing a diverse range of novel materials, including bulk, thin films, monolayers, etc., for their general physical properties^1–4^. To find viable candidates for next-generation technologies, material projects are gradually created within the commitment of characterization and information extraction criteria^5^. Over the last few decades*,* researchers have picked up massive interest in perovskite oxides (both simple and complex) due to their physically intriguing characteristics, particularly their half-metallicity as ferromagnetic half-metallic or ferrimagnetic half-metallic materials devoted to spintronics; besides, the Curie temperature of these compounds has been realised to be exceptionally close to the ambient temperature^6,7^.

Perovskites with an ABO_3_ stoichiometric structure have continued to receive renewed interest because of their countless potential benefits, including the ability to accommodate the majority of elements from the periodic table and their straightforward, rapid, easy, and cost-effective manufacturing process^8–10^. Recent research has mostly centred around this family of compounds due to their adaptable chemical makeup, versatile crystal structure, the possibility of tweaking electronic and magnetic properties, and other impactful physical and chemical features, which has kickstarted a genuine interest in the applied fields of material sciences^11–13^.The variability in their electronic structures presses the materials to vary in behaviour, ranging from metallic to insulating and even to associated half-metallic to spin polarisation, resulting in a plethora of half-metallic materials like ferromagnetic (FM), ferrimagnetic (FiM), and antiferromagnetic (AFM) with significant responses in diverse technological domains. Half-metallic ferromagnet (HMF) compounds, first anticipated by De Groot et al.^14^, are currently the most appealing materials in this decade since they exhibit exceptionally unique electronic structures in which one spin channel exhibits a metallic character, backed by the majority of metallic spin electrons, and the other spin channel reflects a semi-conducting/insulating character, aided by the marginal spin electrons. The electronic density of states (DOS) of HM materials expresses absolute (100%) spin polarisation at the Fermi level, and the conductivity in such materials is overseen by the bulk of metallic spin electrons. Half-metallic materials have been an impeccable option for sophisticated spintronics since they are designed to improve the efficiency of spintronic devices, which do have substantial advantages over conventional electronics in accordance with lower energy consumption, improved circuit integration density, and faster data processing speed^7,13^. This initiative has resulted in extensive theoretical and experimental research on a wide range of materials, including the perovskite alloys La_0.7_Sr_0.3_MnO_3_^15^ and NdGaO_3_^16^, the double-perovskite Sr_2_FeMoO_6_^17^, the metal oxides Fe_3_O_4_^18^ and CrO_2_^19^; Mn and Cr-doped ZnTe, and V-and Cr-doped GeTe^20^; and heusler compounds (Co_2_Ti_1−x_Fe_x_Ga)^21^. Among all, perovskite oxides with ABO_3_ structural arrangements, notably *f*-electron-based, have been widely acknowledged for unveiling the half-metallic character as a consequence of the interactions of localised *f*-electronic states at the Fermi level, resulting in an intriguing electronic profile with strong spin polarisation, significant magnetism, and high phase transition temperature, posing better advancements in sophisticated spintronics. They also demonstrate other unrivalled features like ferroelectricity, superconductivity, ion conductivity, piezoelectricity, and incredible magnetoresistance^22,23^. Recently, Khandy et al. and Dar et al. investigated a series of materials from the perspective of an *f*-electron system (BaNpO_3_^24^, BaPuO_3_^25^, and SrPuO_3_^26^), and they came up with the conclusion that these compounds have a decisively half-metallic nature. Further comprehensive studies concerning *f*-electron-based perovskites have centred both on single and double perovskites, with the majority of the investigated perovskites documented as half-metallic ferromagnets holding dominating *f*-electronic states at the Fermi level, prompting them to be viewed as potential spintronic prospects^27–30^.

On the other hand, the scientific community in the present era is dealing with two major energy-related challenges. The first is the present energy crisis, and the second is its impacts on the environment as a direct consequence of the traditional approaches to using energy resources. These concerns have stimulated research into alternative energy sources and pushed for more innovative and efficient ways to utilise the existing energy sources. Thermoelectricity and its astounding capability to transform waste heat into useful energy are seen as one of the potential solutions to both these concerns. TE materials are likewise beneficial for smart sensors, energy harvesting, and the newly proposed thermo-power wave sources^31^. Perovskites are typical thermoelectric materials that will recreate a key role in thermoelectric devices in the future^32,33^. Many rare-earth-based perovskites have undergone extensive theoretical^22,23,29,34–39^ and experimental^40–42^ scrutiny to comprehend a deeper understanding of their thermoelectric accomplishments. For most of the surveyed rare-earth materials, typically *f-*electron-based, such as SrMnO_3_^34^, GdMn (Tb)O_3_^35^, Sr_2_HoNbO_6_^36^, Sr_2_ReEuO_6_^37^, the optimum thermoelectric output, as decided by the figure of merit (_Z_T), has been revealed. Likewise, phenomenal seeback coefficient and power factor values have been captured for many other *f-*electron-based materials (XVO_3_ (X = Rb, K, Na)^38^, Ba_2_MgMO_6_ (M = Np, U)^39^, BaBkO_3_^22^, BaREO_3_^23^, Ba_2_MUO_6_ (M = Co, Fe)^29^). All of these literature findings emphasize the significance of *f*-electron-based materials being well suited for thermoelectric technology. The better thermoelectric performance of *f*-electron-based perovskites lends their route in radioisotope thermoelectric generators (RTG) applications^43,44^. These devices (RTG) operate like other thermoelectric devices and generate electricity with the assistance of the Seeback effect; nevertheless, the fuel used in RTG devices is tendered by the decay of radioactive material. Novel materials with high thermoelectric output must be probed to maximize the performance of RTG solid-state devices. Put bluntly, *f*-electron-based perovskites, branded as multifunctional materials, offer potential applications in several advanced technological domains, as discussed above, prompting us to perpetrate the present research work.

The present study investigates the structural parameters, spintronic properties, and thermoelectric response of actinide-based RbMO_3_ oxides with the possible intention of identifying novel (wide band gap) half-metallic materials for spintronics and thermoelectric device applications. The substituted forms of comparable materials like BaNpO_3_, BaPuO_3,_ and SrPuO_3_ have been investigated both theoretically and experimentally. These investigations, however, were incapable of clarifying the thermoelectric behaviour, thermodynamic stability, Curie temperature, and other pertinent physical features of these materials. Herein, for the first time, we have made a successful computational attempt to figure out the structural, spintronic, and transport properties of *f*-electron-based RbMO_3_ perovskites. The occurrence of these materials has been predicted by Xiang et al.^47^, and the available literature asserts their stability by expressing their tolerance coefficient and stability index factor^47^. In addition, Haiying Liu et al.^48^ conveyed the convex hull energetics of these alloys, reflecting their thermodynamic stability. As a consequence, these aspects have driven us to investigate their ground-state properties for possible applications in device fabrication. To the best of our knowledge, no research findings in the scientific literature have yet looked at the ground-state properties and potential applications of these oxides. Hence, we performed DFT-based simulations to thoroughly examine the structural stability, electronic profile, transport coefficients, and mechanical stability of RbMO_3_ (M = Np, Pu) perovskites.

## Method of calculation

All the simulations in this work were accomplished within the framework of the full-potential Wien2k program^49^. In view of the Kohn–Sham equations, we simulated the ground-state electron density of the given perovskites, employing a well-known generalized gradient approximation (GGA) under Perdew, Burke, and Ernzerhof parameters to approximate exchange–correlation (*E*_*xc*_) interactions^50^. In addition to the GGA, the more accurate exchange–correlation potential known as modified Becke–Johnson (TB-mBJ) has been implemented to facilitate the results obtained through GGA approximations to be more accurate^51^. The Tran–Blaha modified Becke–Johnson (TB-mBJ) potential consists of an LDA potential for correlation and an altered BJ potential for exchange, which precisely replicates the KS potential of atoms. The exchange part, which is a meta-GGA (MGGA) since it is impacted by the kinetic-energy density t, is given as;$v_{x,\sigma }^{TB - mBJ} (r) = cv_{x,\sigma }^{BR} (r) + (3c - 2)\frac{1}{\pi }\sqrt{\frac{5}{12}} \sqrt {\frac{{2t_{\sigma } (r)}}{{\rho_{\sigma } (r)}}}$ where ${V}_{x,\sigma }^{B,R}$ is the Becke–Roussel (BR) potential and $c = \alpha + \beta \left( {\frac{1}{{V_{cell} }}\int {\frac{{|\nabla \rho (r^{^{\prime}} )}}{{\rho (r^{^{\prime}} )}}} d^{3} r^{^{\prime}} } \right)^{\frac{1}{2}}$. The parameters α =  − 0.012, β = 1.023 bohrs^1^^/^^2^ are presumed by minimising the mean absolute error of the bandgap for a group of solids. The incorporation of the mBJ potential produces computationally more efficient results and is currently the most accurate semi-local method for band gap prediction^22,24,29^. Further specific details regarding other parameters involved in the present study are provided in the supplementary information file. The thermoelectric response has been explored involving semi-classical Boltzmann theory as executed in the BoltzTraP code^52^ and appended with the Wien2k program. Calculations based on elastic constants have been carried out to investigate the mechanical behaviour in accordance with Charpin's Cubic-elastic package^53^. The thermal properties are evaluated using the Gibbs2 package in aggregation with the Wien2k code^54^.

**Figure 1:**
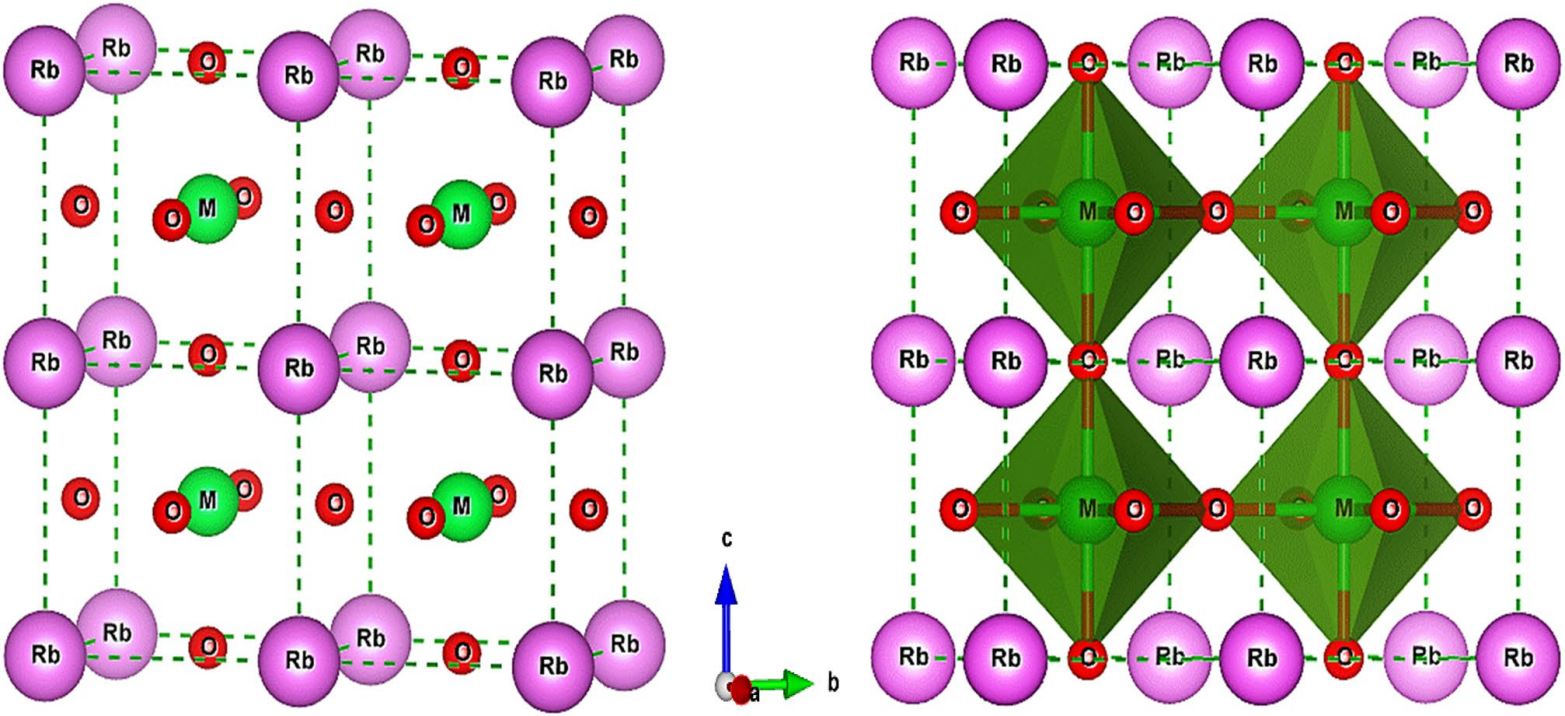
The layout of various atoms in a conventional unit cell (left); and the crystal structure of RbMO_3_ perovskites (right).

After rigorous investigations, we find that RbMO_3_ perovskites correspond to the Pm-3 m (221) cubic space group, where the monovalent Rb cations are 12-fold coordinated by oxygen anions and take up the corner positions (0, 0, 0). The pentavalent M (Np, Pu) cations lie within the oxygen octahedral occupying the body centre positions (0.5, 0.5, 0.5), and the oxygen atoms hold the face centre positions (0.5, 0.5, 0) of the cubic lattice. The placement of these atoms in the cubic unit cell is shown in Fig. 1 (left), and the cubic structure of RbMO_3_ perovskites, flaunting a 3-D network of corned MO_6_ octahedra, is pictured in Fig. 1 (right).

## Results and discussion

The results and discussion of expected physical (structural, electronic, mechanical, and thermal) and transport properties are outlined in the sections below.

### Geometric structure, volume optimization and thermodynamic stability

As a first step, it is critical to comprehend the structural stability of the materials under discussion. Generally, perovskites with an ABO_3_ stoichiometry display a flawless cubic crystal structure. However, size effects and octahedral tilt can trigger structural deformities within the perovskite structure; these disfigurements are explicitly intervened by (i) B ion displacements in the BO_6_ octahedron, (ii) tilting of the BO_6_ octahedron, and (iii) Jahn–Teller distortions. To expect minimum structural deformations and, consequently, cubic stability of RbMO_3_ perovskites, we have recourse to the Goldschmidt tolerance and the octahedral factor, which are excellent predictors of the stable perovskite structure. The tolerance factor tackles the size-effect parameter in ABO_3_ crystals, though the octahedral factor characterizes their overall stability, and both were evaluated using the following equations^55,56^;

$$t_{G} = \frac{0.707 (A-O)}{(B-O)} \quad \quad (\mathrm{Goldschmidt} \, \mathrm{method})$$

$$t=\frac{0.707({r}_{A}{+r}_{O})}{({r}_{B}{+r}_{O})} \quad \quad(\mathrm{Ionic} \, \mathrm{ method})$$

$$\mu =\frac{{r}_{B}}{{r}_{O}} \quad \quad(\mathrm{Octahedral} \, \mathrm{ factor})$$

Here, the symbols A–O and B–O signify the mean bond lengths of the atoms Rb, O, and Np/Pu, respectively, while r_A_, r_B_, and r_O_ are counterparts to the ionic radii of Rb, Np/Pu, and O atoms. The structural stability of a perovskite structure is supposed to be directed respectively by the tolerance and octahedral factor, which must be between 0.813 < t < 1.107 and 0.377 <  μ < 0.895^55,56^. The derived values for tolerance and octahedral factor are summed up in Table 1. The tolerance parameter estimated by both methods approaches unity, asserting the cubic stability of RbMO_3_ compounds, besides making sure that the MO_6_ octahedron does not present any tilt to take up space in the crystal. Likewise, the reasonable values of the octahedral factor, which are higher than the critical value (0.337), further ascertain the structural stability of the studied compounds. Consequently, the present perovskites crystallise in the Pm-3m (221) space group, with Rb, M, and O atoms positioned at 1a (corners), 2b (body centre), and 3c (face centre) Wyckoff coordinates following the symmetry *m*3*m, m*3*m*, and 4*/mmm*, as displayed in Fig. 1. The well-ordered cubic structure of RbMO_3_ compounds exhibits a corner-linked (distortion-free) MO_6_ octahedra connectivity, with all M–O–M angles equaling 180 degrees. As of now, this is the first simulation-based theoretical and quantitative investigation of these compounds; experimental validation is still awaiting to substantiate theoretical results.

**Table 1:** Illustrated values of the relaxed lattice parameter ($${a}_{0})$$, bulk modulus (*B*), pressure derivative of bulk modulus (*B*'_0_), ground state energy (E_0_), tolerance factor (*t*), octahedral factor (μ), (cohesive energy (E_coh_), energy of formation ($$\Delta H$$) and critical radius (r_c_) of cubic RbMO_3_ compounds.

Compound	${a}_{0}$ (Å)	*B* (GPa)	*B*'_0_	E_0_ (eV)	*t *(ionic)	Bond lengths		*t*_*G*_	μ	E_coh_ (eV)	$\Delta H$ (eV)	*r*_*c*_
RbNpO_3_						Rb–O	Np-O					
Present	4.33	129.8	4.55	− 872,767.845	1.00	3.06	2.16	1.00	0.56	23.6	− 2.38	0.70
Analytical	4.28											
Theory	4.43^a^ 4.37^b^	128.9^a^ 117^b^	3.17^a^ 4.77^b^							22.4^g^		
Exp	4.38^c^											
RbPuO_3_												
Present	4.32	126.4	4.73	− 894,867.971	1.00	3.15	2.23	0.99	0.55	22.8	− 2.19	0.75
Analytical	4.27											
Theory	4.39^d^ 4.33^e^	126^d^ 120^e^	3.87^d^ 5.8^e^							22.5^g^		
Exp	4.38^*f*^											

[a]^24^, [b]^28^, [c]^45^, [d]^25^, [e]^26^, [f]^46^, [g]^13^. The results of other perovskites^13,24–26,28,45,46^ are listed for comparison.

As a second step, it is a question of determining the lattice constant to simulate the given compounds. The lattice constant can be chosen systematically using the relation^57^; $a=\alpha +\beta ({r}_{Rb}$+${r}_{O}$) + $\gamma ({r}_{M}$+${r}_{O}$), here $\alpha$ (0.06741), $\beta$ (0.4905) and $\gamma$(1.2921) are numerical constants, while ${r}_{Rb}$, ${r}_{M}\mathrm{ and }{ r}_{O}$ reflects the ionic radii of Rb (1.72 Å), Np (0.75 Å), Pu (0.74 Å***)*** and O (1.35 Å), respectively. The calculated values of the analytical lattice constant of both compounds are outlined in Table 1. Subsequently further, utilizing the analytical lattice, the present materials were simulated in FM, AFM, and NM magnetic configurations to extract the ground state structural parameters by performing a least-squares fit of the crystal energy against the unit cell volume through the Birch-Murnaghan equation of state^58^;

$$E(V) = E_{0} + \frac{{9B_{0} V_{0} }}{16}\left\{ {\left[ {\left( {\frac{{V_{0} }}{V}} \right)^{2/3} - 1} \right]} \right.B^{\prime}_{0} + \left[ {\left( {\frac{{V_{0} }}{V}} \right)^{2/3} - 1} \right]^{2} \left. {\left[ {6 - 4\left( {\frac{{V_{0} }}{V}} \right)^{2/3} } \right]} \right\}$$

**Figure 2:**
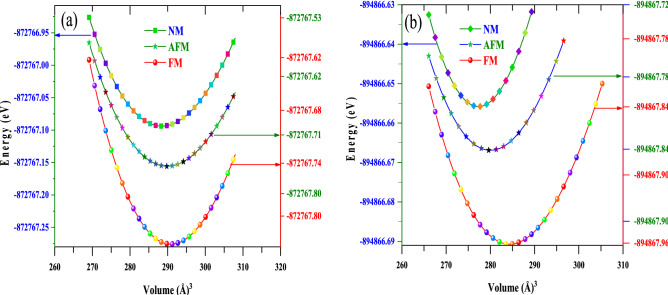
The optimised energy-volume plots for (**a**) RbNpO_3_ and (**b**) RbPuO_3_ oxides in three different magnetic phases (FM, AFM, NM), determined via the PBE-GGA approximation.

The terms *E*(*V)*, *V*, and *B*_*0*_ (*B'*_*0*_) in this equation connote the ground state energy, unit cell volume, and the bulk modulus (pressure derivate of the bulk modulus), respectively. Among these, the bulk modulus(*B*_0_) and pressure derivative of the bulk modulus(*B'*_0_) are the two decisive parameters that convey significant material characteristics and are methodically defined as:$B_{0} = - V\left( {\frac{\partial P}{{\partial V}}} \right)_{T}$ and $B^{\prime}_{0} = \left( {\frac{\partial B}{{\partial V}}} \right)_{T}$. The pressure *P* in this instance is the negative derivative of total energy. Hence, bulk modulus precisely quantifies the curvature of E–V curve. Bulk modulus, in general, gauges a material's capability to withstand volume changes when pressurised from all sides. Moreover, it also serves as an excellent predictor of the hardness and rigidity of a material. For RbNpO_3_ and RbPuO_3_, the observed values of bulk modulus have been recorded as 129.8 (GPa) and 126.4 (GPa), respectively, which are relatively higher than other reported perovskites (Table 1), reflecting the more rigid nature of the given compounds. The pressure derivate of bulk modulus (*B*'_0_), on the other hand, truly depicts the rate at which bulk modulus expands in response to increments in pressure. It is a dimensionless parameter that defines the curvature of the bulk modulus under pressure and makes the study of the systematics of *B*_0_ very fascinating. It should be noted that the pressure derivative of bulk modulus is of central importance for evaluating the thermoelastic properties of materials at high pressures and high temperatures. The AFM calculations for the two compounds were executed by assessing the AFM-G phase with the spin configuration of the atoms assigned by default within the AFM program. The optimized energy-volume plots for RbMO_3_ oxides, as illustrated in Fig. 2a and b, spell out all other magnetic layouts by announcing that the FM phase, which holds the least energy, has been the most stable magnetic phase. The positive energy difference, $\Delta E=\left(AFM-FM\right),$ asserts the increased stability of the FM state for the given compounds. The structural ground state parameters such as relaxed lattice constant $({a}_{0})$, ground-state energy (E_0_), bulk modulus (*B*_0_), and its pressure derivative (*B*'_0_) have been listed in Table 1. All these parameters are in fine agreement with previously published computational and experimental findings, as shown in Table 1, corroborating our results.

Further authenticity regarding thermodynamic concerns is sought for structural stability as the alloys under study have not yet been experimentally synthesized*.* The thermodynamic stability for the given compounds has been determined using cohesive and formation energy calculations, which are discussed in the supplementary information file. The values intended for these entities are listed in Table 1. Based on the cohesive energy estimate, the present alloys are stable, and the atoms in their appropriate lattice positions are tightly bound, preserving these structures over a diverse range of forces. Also, the cohesive energy totals inserted in Table 1 are comparatively higher than previously reported perovskites, justifying the more stable energy state of the given materials. Remarkably, the negative enthalpy of formation for these compounds indicates that they can be produced experimentally and are likewise energetically stable.

Furthermore, the critical radius (r_c_), a significant structural parameter that impacts the synthesis of perovskites, has also been calculated for the given materials and is covered in the supplementary information file.

### Electronic and magnetic profile

#### Band profile

**Figure 3:**
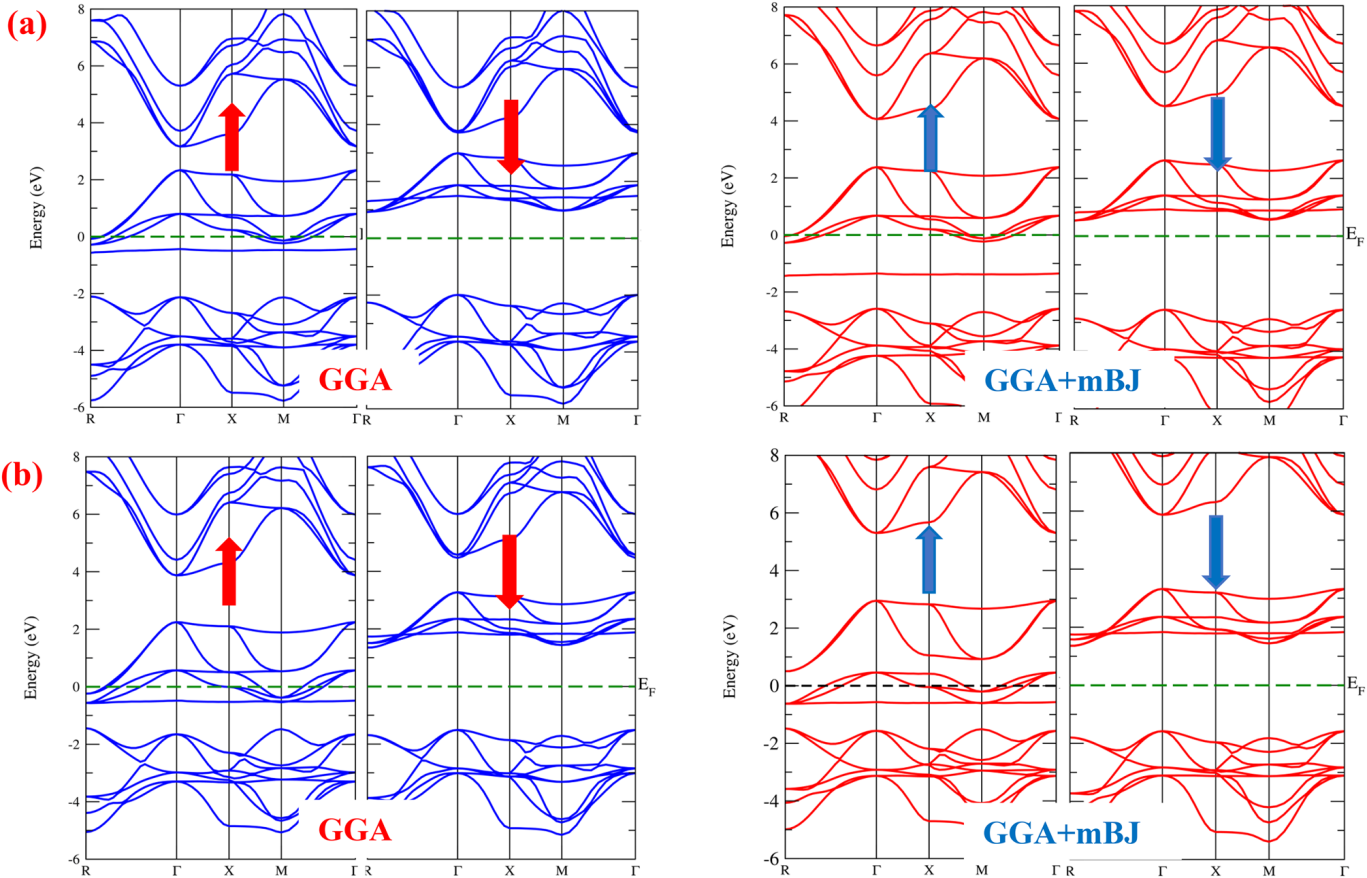
Spin polarised electronic band structure of cubic (**a**) RbNpO_3_ and (**b**) RbPuO_3_ reckoned by PBE-GGA and GGA + mBJ approximations.

The spintronic properties of the given compounds were analysed by evaluating the electronic band structure (BS) and density of states (DOS) using self-consistent spin-polarized calculations. The electronic band structure (BS) critically examines magneto-electronic and thermoelectric properties of a material at the subatomic scale; henceforth, a methodical description of the band structure is meant to conceive authentic paradigms of these properties. The bandgap, a significant component of the band structure, characterizes the nature of a material and hence should be precisely evaluated. Herein, the mBJ approach has been adopted to properly assess the band gap since it supplies energy band gap values that closely align with experimental results^51^. The electronic band structures (BS) of cubic RbMO_3_ compounds have been determined by exploiting the *k*-momentum points following the path *R − T − X − M − Γ* for spin-up and spin-down states in the irreducible Brillouin zone. The retrieved band structures are depicted in Fig. 3a and b for GGA and GGA + mBJ approximations, respectively; inferring that the electronic band structure (BS) unveils metallic behaviour for the spin-up state because of the decisive Fermi level overlap of the valence and conduction bands. This Fermi level overlap, and subsequently the metallic nature in the spin-up state, could be credited to electronic states that exclusively emerge from 5*f*-Np/Pu localised states as they descend over the Fermi level. Conversely, the spin-down state divulges semiconducting nature as the confined 5*f*-Np/Pu states are driven deep inside the conduction band, letting in a gap between the corresponding band edges (VBM and CBM). The overall band structure reflects 100% spin polarisation at the Fermi level, accomplishing the half-metallic nature of both materials. These compounds, therefore, uphold a fully spin-polarized current, making them excellent prospects for spin injection applications in sophisticated spintronic devices. Further, a glancing look at the electronic band structure deduces band gap perceptions, as can be traced in Fig. 3a and b, where the specific band edges (VBM and CBM) for the down spin state are sited at two distinct symmetry points, which typically results in an indirect band gap value of 2.94 eV and 2.90 eV for RbNpO_3_ and RbPuO_3_, respectively, in the GGA approximation. However, the band gap improves precisely when the mBJ potential gets involved, attaining values of 3.10 eV for RbNpO_3_ and 3.04 eV for RbPuO_3_, respectively. The band gap in GGA + mBJ potential expands as the energy bands favoured by Np/Pu-*f *states are swept further away from the Fermi level. The overall band structure reflects the half-metallic behaviour, with metallic spin-up and semiconducting spin-down states. The comparable findings have been reported by Khandy et al.^24^ and Dar et al.^25^, as illustrated in Table 2. While looking at the findings, we might assert that the present compounds flaunt a lower band gap than other reported materials^13,24,25^, pressing them as better prospects for thermoelectric applications. Nonetheless, the band gap values exceed 2 eV, classifying them as wide bandgap semiconductors capable of operating at higher temperatures, frequencies, and voltages than traditional semiconductor materials such as silicon. Furthermore, significant pseudo gaps are witnessed in the entire band structure. The half-metallic gap, also known as the spin-flip gap, predicts the minimum energy needed for an electron to hop from the highest point of its occupied valence to the Fermi level and is computed as the distinction in energy between the VBM and the Fermi level^6^. The calculated values of the HM gap are specified in Table 2. The identified HM gap values are higher than other comparable perovskites reported in the literature. Thus*,* we can speculate that the given materials have a wider HM gap and could be considered potential half-metallic ferromagnets for advanced spintronics.

**Table 2:** Illustrated values of indirect band gap (eV) and half-metallic gap (eV), magnetic moment (µ_B_), determined via GGA and GGA + mBJ approximations for RbMO_3_ perovskites.

Parameter	Band gap		Half-metallic gap		Magnetic moment		Nature of band gap	Nature of electronic structure
Method	GGA	mBJ	GGA	mBJ	GGA	mBJ	GGA + mBJ	GGA + mBJ
RbNpO_3_	2.94	3.10	1.96	2.54	2.00	2.00	Indirect	HM
RbPuO_3_	2.90	3.04	1.50	1.57	3.00	3.00	Indirect	HM
BaNpO_3_	3.7^a^	3.8^a^	**–**	**–**	3.00^a^	3.00^a^	Indirect^a^	HM^a^
BaPuO_3_	3.4^b^	3.8^b^	**–**	**–**	4.00^b^	4.00^b^	Indirect^b^	HM^b^
BaMgO_3_	5.6^c^	7.6^c^	0.49^c^	0.62^c^	2.00^c^	2.00^c^	**–**	HM^c^
BaCaO_3_	5.1^c^	7.1^c^	0.58^c^	0.60^c^	2.00^c^	2.00^c^	**–**	HM^c^

[a]^24^, [b]^25^, [c]^13^. The findings of other perovskites^13,24,25^ are listed for comparison.

#### Density of states

**Figure 4:**
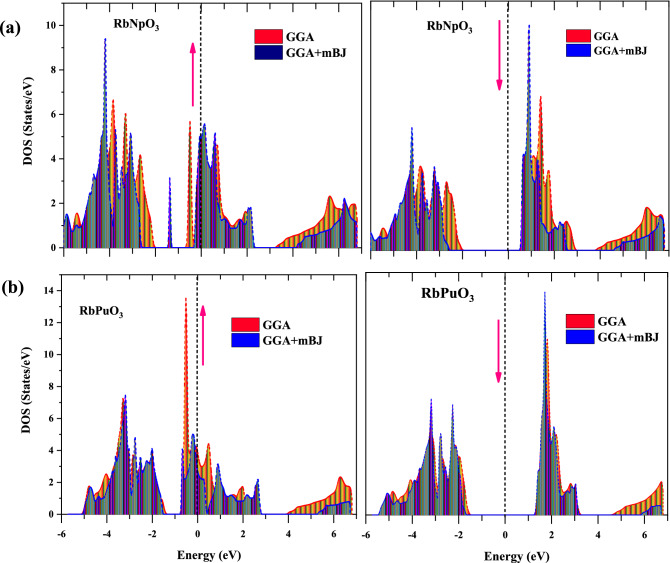
Total density of states (TDOS) predicted by GGA and GGA + mBJ approximations for (**a**) RbNpO_3_ and (**b**) RbPuO_3_ compounds.

**Figure 5:**
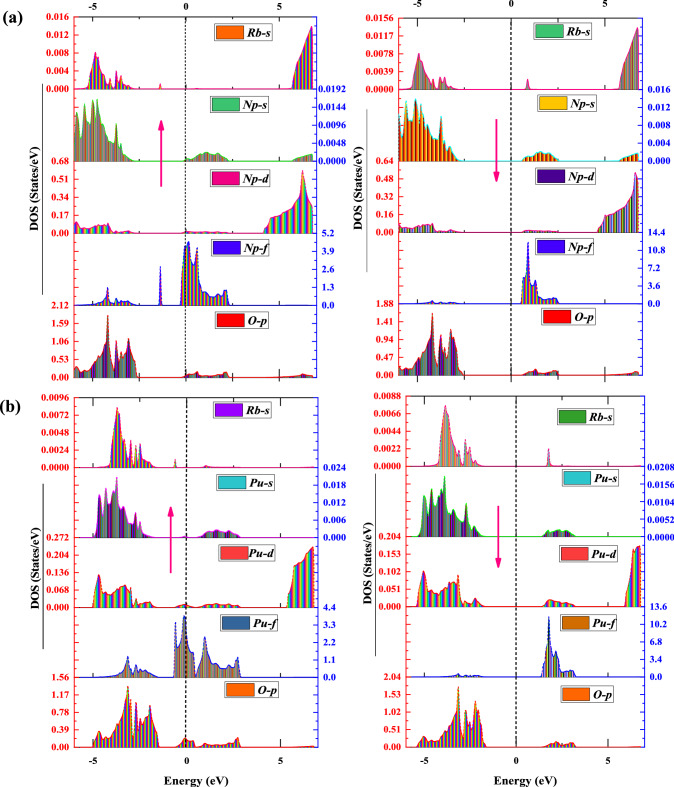
Partial density of states (_P_DOS) forecasted via GGA + mBJ approximations for (**a**) RbNpO_3_ and (**b**) RbPuO_3_ compounds.

Although the electronic band structure (BS) encapsulates a material's electrical properties, it lacks a comprehensive interpretation of the permitted energy levels of various particle states. Thus, to envision the adequate treatment of the electronic profile, we must proceed to the density of states (DOS), which eases us to compute the overall distribution of states in response to energy. Also, the DOS parameter greatly impacts the bulk properties of conductive materials like heat capacity, magnetization susceptibility, and other transport processes. The total density of states (TDOS) has been predicted to gain insights into the contributions of different particle states in the electronic band structure, and the gotten results are displayed in Fig. 4a and b using the GGA and GGA + mBJ parameterizations, respectively. The observed TDOS graphics deliver further confirmation of the half-metallic nature quantified by the electronic band structure (BS). In addition, the projected density of states (PDOS) has been adopted to assess the selective contribution of each atom to the total DOS for RbNpO_3_ and RbPuO_3_ in their respective spin up and spin down states within the scenario of the GGA + mBJ scheme, as shown in Fig. 5a and b. The Fermi level in the DOS spectrum is fixed at zero energy, partitioning the entire energy zone into two distinct sections. The region below the Fermi is known as VB, while the region above it is called CB.

For RbMO_3_ perovskites, Rb (*6s*^*1*^), Np (*7s*^*2*^*,6d*^*1*^*5f*^*4*^), Pu (*7s*^*2*^*, 6d*^*0*^*,5f*^*6*^*,*) and O (2*p*4) are treated as valence states. The commitments of the orbital contributions of involved states Rb-s, Np/Pu-s, Np/Pu-d, Np/Pu-*f*, and O-*p* to the band composition are shown in Fig. 5a and b. Of all the states, the *f*-states of Np/Pu are the most intriguing states since they reside close to the Fermi level. The O-*p* states that make up the majority of the VB receive electrons from cations (Rb^+1^ and Np^+7^/Pu^+8^). As displayed in Fig. 5a and b, *Rb-s*, *Np/Pu-s*, and* Np/Pu-d* states are nowhere near the Fermi level, so they have a negligible impact on the overall electronic profile of these perovskites. While interpreting the _P_DOS for the RbNpO_3_ compound, we find Np-5*f* states overtake both the VB and CB regions in the spin-up state between a certain energy range of − 5 to 2 eV, with significant contributions from O-2*p* states residing in the energy range of − 6 to − 2 eV. Interestingly, the partially filled Np-5*f *states in the up-spin configuration exceed the Fermi level, disclosing the metallic behaviour of the RbNpO_3_ compound. However, in the spin-down state, O-2*p* states captured between − 6 and − 2 eV intensely hybridize with Np-5*f* states, mopping them away from the Fermi level elsewhere in the energy range of 0.8–2.5 eV, which consequently results in the onset of the band gap. Meanwhile, spin-down states relocate deep inside the conduction band in rebuttal to exchange energy, contributing to the bandgap widening and thus adopting semiconducting behaviour. The overall _P_DOS sketches the half-metallic character of RbNpO_3_ perovskite. To conclude, it is imperative to stress the significance of robust hybridization of Np-5*f *with O-*p* states in deciding the half-metallic character of the cubic RbNpO_3_ compound. The _P_DOS for another compound, RbPuO_3_, typically follows a similar pattern.

### Electronic charge density behaviour

**Figure 6:**
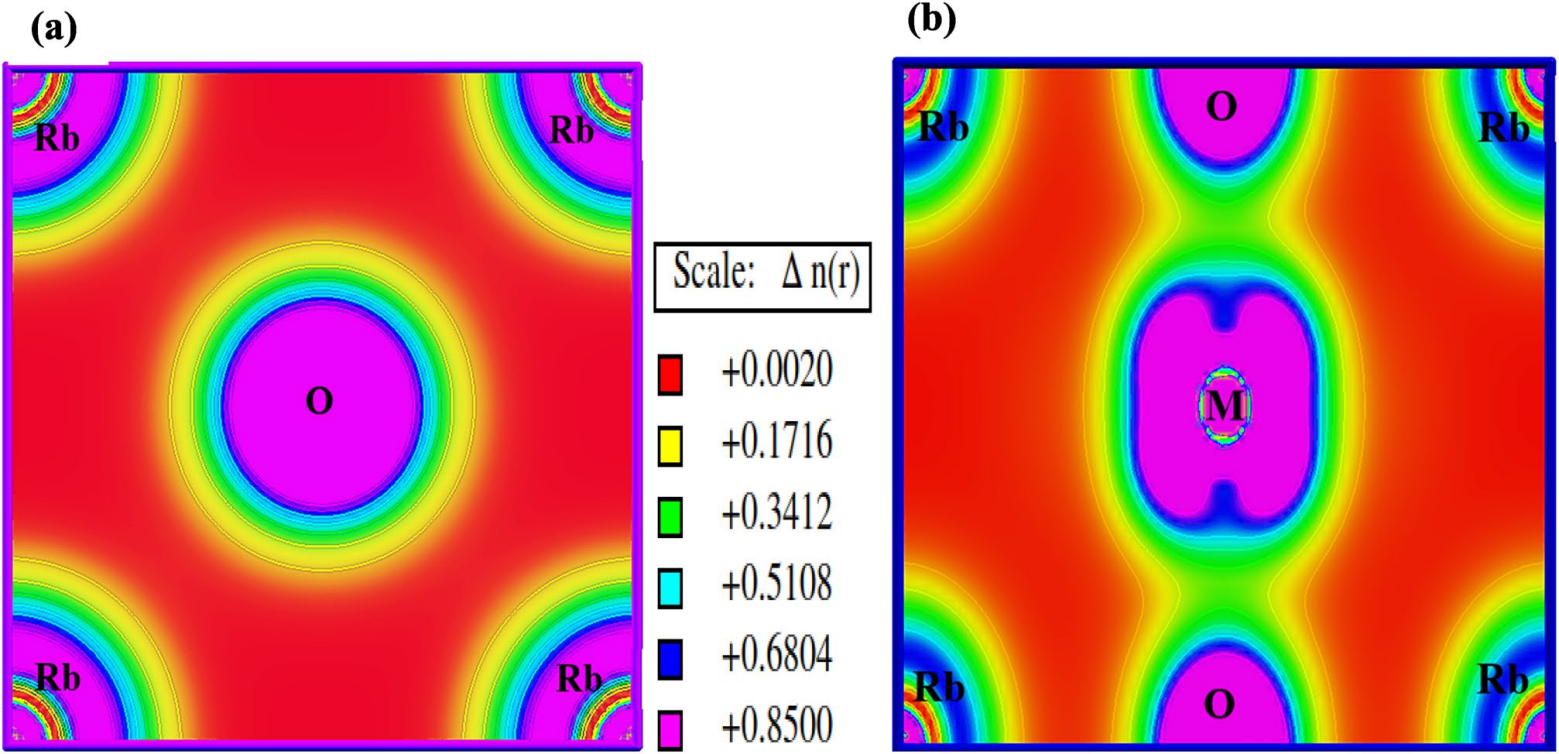
Electronic charge density distribution in RbMO_3_ perovskites along (**a**) 100 and (**b**) 011 planes.

The constituent atoms which make up a crystal are interlinked by bond linkages that hold unique spatial charge distributions. Covalent and ionic bonds are two different types of bond connections. The ionic bond, which reveals the sole transfer of electrons from one atom to another, exhibits negligible charge density across the interatomic distances, in contrast to the covalent bond, which has a non-zero density throughout the bond distance. To better comprehend the bonding properties of RbMO_3_ perovskites, charge density plots were projected along the 100 and 011 planes, as illustrated in Fig. 6a and b. These plots reflect that the corresponding atomic sites capture the maximum charge impelled by the respective atoms. The dissemination of electronic charge clouds between the Rb and O atoms is inherently spherical (Fig. 6a, asserting that the Rb atom invests 100% of its time in the proximity of oxygen; this backs up the possibility of an ion bond between the two atoms, which is further aided by an electronegativity difference of magnitude 2.57 on the Pauling scale that appeases the ionic bond formation between them. However, upon closer look of the charge clouds along M (Np, Pu)-O (Fig. 6b, dumbbell-shaped clouds rather than spherical clouds appear, reflecting the 50%-time devotion of Np/Pu in the vicinity of oxygen and thus disclosing the covalent bond between Np/Pu and O atoms. Also, these dumbbell-shaped clouds along M (Np, Pu)-O demonstrate a sizable overlap between Np/Pu and oxygen atoms, correlating with DOS plots that deep-rooted a robust hybridization between 5*f* states of Np/Pu and *p*-states of oxygen atoms. The shifts in bonding behaviour might be attributed to the increments in electronegativity across Np and O atoms. As a whole, the polar covalent bonding, which is a mix of ionic and covalent bonds, is preserved within these lattice structures.

### Magnetism and Curie tempertaure

The most critical factors in shaping a material's magnetism are its electronic profile and ground-state magnetic phase. The ferromagnetic ground state has been perceived as the stable magnetic phase by total energy-volume calculations. Also, the electronic profile clarifies that the *f*-orbitals of Np and Pu states contribute the most to the electronic states around the Fermi level. Hence, these states are indispensable for leveraging the total magnetic moments of RbMO_3_ perovskites. The total magnetic moment assumes the aggregate of orbital and spin magnetic moments; however, due to the quenching of orbitals in highly correlated systems, only the spin magnetic moments are frequently measured. The spin magnetic moment of any material is calculated as^59^; ${\mu }_{s}=\sqrt{4S(S+1)}$= $\sqrt{n(n+2{)}_{{\mu }_{B}}}$, here the total spin and number of unpaired electrons are expressed as *S* and *n*, respectively. In line with the present compounds, RbNpO_3_ has two unpaired electrons, though RbPuO_3_ has three, giving credence to total spin values of *S* = 1 for RbNpO_3_ and *S* = 3/2 for RbPuO_3_, respectively. The spin magnetic moments of RbNpO_3_ and RbPuO_3_ are expected to be 2.82 ${\mu }_{B}$ and 3.87 ${\mu }_{B}$, respectively. Nevertheless, the calculated spin magnetic moments for RbNpO_3_ and RbPuO_3_ in the GGA and GGA + mBJ methods respectively have integral values of 2 ${\mu }_{B}$ and 3 ${\mu }_{B}$. The lower magnetic moments than expected could be linked to strong hybridization effects, which are fully accountable for shifting some magnetic moments from Np/Pu atoms in their free space values to non-magnetic ions like oxygen and interstitial sites. The total spin magnetic moments and the findings of other comparable reported perovskites are in close agreement, as outlined in Table 2. The positive integral spin magnetic moment further certifies the half-metallic nature of the representative perovskites. The high spin magnetic moments offer better advancements of these materials for MRAM applications in spintronics, where magnetic moments rather than electrical charges are used to store information^60^.

#### Curie temperature

A ferromagnetic or ferrimagnetic substance forfeits its permanent magnetization at a temperature known as the Curie temperature (T_*C*_). In the present study, the Curie temperature was estimated theoretically within the mean-field approximation (MFT), based on the Heisenberg model. In such approximation, the Curie temperature for magnetic systems is calculated as^6,61^; ${T}_{C}=\frac{2\Delta E}{3x{K}_{B}}$, where $\Delta E$ is the fundamental parameter in the Heisenberg model of the mean-field approximation for predicting magnetic transition temperature and ${K}_{B}$, which explicates the relation between the temperature and energy level, stands for the Boltzmann constant. Following the calculations, the Curie temperature for RbNpO_3_ and RbPuO_3_ was projected to be 309.46 K and 464.19 K, respectively. The predicted values of the Curie temperature are higher than those of previously reported perovskites^6^ (295 K, 290 K, 285 K, 277 K) which may be due to pretty $\Delta E$ parameters and considerable magnetic moments for the present compounds. The high curie temperature legitimizes stronger interactions among magnetic ions as well as guarantees that these compounds preserve ferromagnetic phase stability much above ambient temperature.

**Figure 7:**
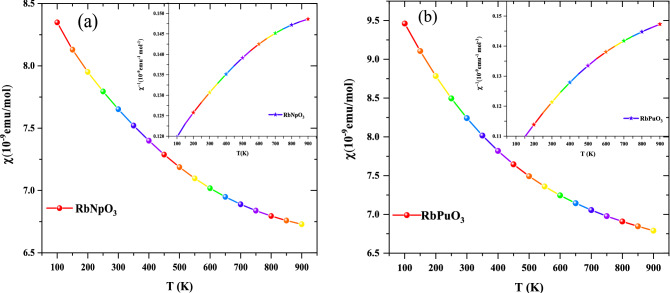
Temperature dependence of magnetic susceptibility (χ) and its inverse (χ^−1^) for (**a**) RbNpO_3_ and (**b**) RbPuO_3_ compound.

The ferromagnetic ground state ascertained by structural optimization plots has further been validated by evaluating the magnetic susceptibility(χ) and inverse susceptibility (χ^−1^) of cubic RbMO_3_ compounds. According to the Curie–Weiss law, a positive value of the Curie-Weiss constant signifies the ferromagnetic interactions, while its negative value depicts antiferromagnetic interactions. Magnetic susceptibility and its inverse have been represented graphically in Fig. 7a and b. The Curie–Wiess constant values for RbNpO_3_ and RbPuO_3_ are estimated to be 100 K and 150 K, respectively*.* The positive values of the Curie–Wiess constant, which are unquestionably much greater than zero, further endorse the ferromagnetic ground state of the present compounds.

### Transport coefficients

Recently, thermoelectric materials have gained significant interest since thermoelectric power generators based on thermoelectric materials have the potential to alleviate the severe challenges of fossil fuel depletion and global warming triggered by an increase in anthropogenic greenhouse gas emissions exacerbated by the combustion of fossil fuels. A significant share of wasted thermal energy can be converted into usable energy by operating highly efficient TE devices (thermocouples, temperature sensors, and power generators). The conversion efficiency of a thermoelectric device is addressed by a dimensionless figure of merit (ZT) given as; $\mathrm{ZT}=\frac{{S}^{2}\sigma }{{\kappa }_{e}+{\kappa }_{l} }\mathrm{T}$, where the symbols, S, σ, κ_e_, κ_*l*_, correspond to the Seebeck coefficient, electrical, electronic and lattice thermal conductivities, respectively. For practical applications of materials in thermoelectrics, ZT ≥ 1. Thermoelectric materials should have eloquent values for the Seeback and electrical conductivity in addition to a low thermal conductivity value to achieve optimal thermoelectric performance. In particular, the unprecedented values of power factor (PF) and figure of merit (ZT) are extremely looked-for. Transport characteristics like the Seeback coefficient, electrical conductivity, and power factor have a pretty interesting association with chemical potential and temperature. The BoltzTraP code^52^ has been utilised to assess the impact of transport coefficients on chemical potential (− 2 < µ > 2) at different temperatures (300 K, 600 K, 900 K) within the constant relaxation time $(\tau =0.5\times {10}^{-15}s)$,which corresponds to the average of all vibrations in the crystal lattice. The constant relaxation time of the carriers has a noticeable effect on the transport coefficients; nevertheless, the constant time approximation works well if fluctuations in ‘$\tau$’ are mild on the energy scale. In the present work, the unavailability of experimental data bars us to compute the relaxation time of carriers since we can’t make fit of the theoretical data. Though, one can forecast the relaxation time using DP theory, which only compensates for acoustic phonons in carrier scattering processes and always exaggerates relaxation time $(\tau )$. Hence, all the transport coefficients have been supplied under the framework of constant relaxation time $(\tau =0.5\times {10}^{-15}s)$ to let experimentalists compare the results directly. The thermoelectric coefficients, such as the Seeback coefficient (S) and total electrical conductivity, have been deduced respectively using two current models, as given by; $S=\frac{S\left(\uparrow \right)\sigma \left(\uparrow \right)+S(\downarrow )\sigma (\downarrow )}{\sigma (\uparrow )\sigma (\downarrow )}$ and $\sigma_{tot}$  = $\sigma \left(\uparrow \right)+\sigma (\downarrow )$*,* where arrows specify the corresponding spin channels^22^. All of the transport coefficients have been illustrated one by one and are listed below.

#### Seebeck coefficient

One of the most key metrics in a thermoelectric study is the Seebeck coefficient (S), which is defined as the proportion of the potential difference generated in response to the applied temperature gradient and is numerically given as; $S=\frac{\Delta V}{\Delta T}$, where $\Delta T$ characterizes the temperature gradient. The generated voltage is strongly impacted by the material's composition and the mobility of its charge carriers. The maximum thermopower may come from charge carriers with just one type of carrier, either p-type or n-type, as mixed carriers lessen the induced potential by counteracting each other's effect.

**Figure 8:**
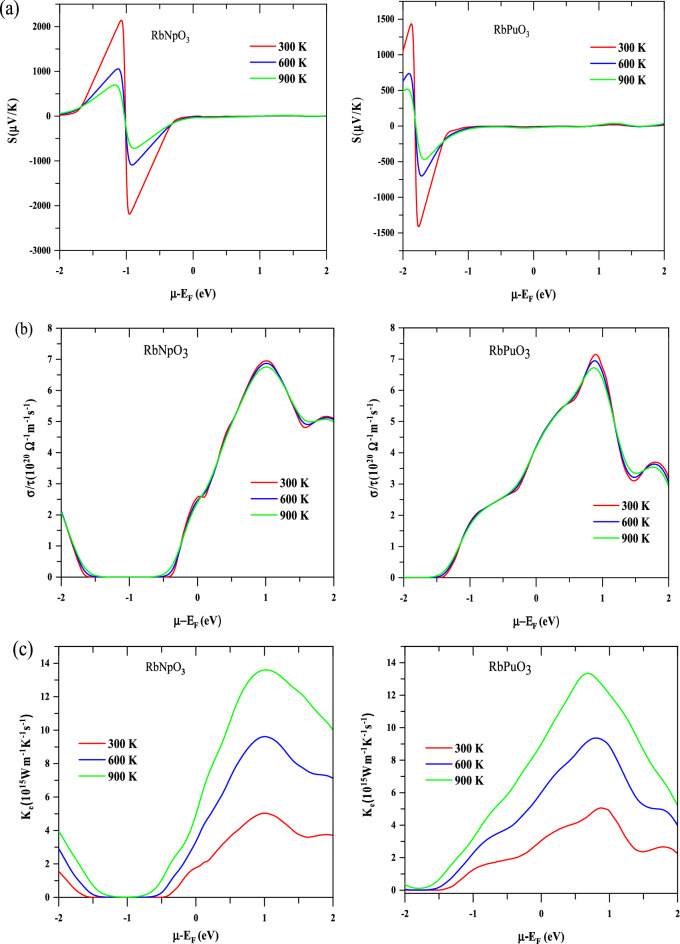

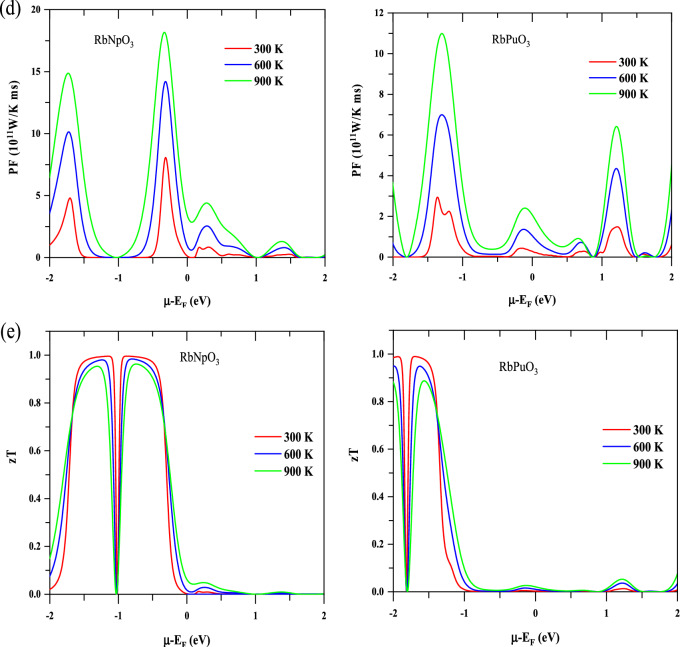
Thermoelectric plots of RbMO_3_ perovskites against chemical potential at various temperatures, viz; (**a**) Seebeck coefficient (S), (**b**) electrical conductivity ($\sigma /\tau$), (**c**) electronic thermal conductivity (${\upkappa }_{e}$), (**d**) power factor (PF), and (**e**) figure of merit (Z_e_T). Temperature is denoted by several colours: red-300 K, blue-600 K, and green-900 K**.**

The total Seebeck coefficient (S) for RbMO_3_ compounds against the chemical potential at different constant temperatures (300, 600, 900 K) has been elucidated, as shown in Fig. 8a; it can be observed that the thermopower reacts quickly to changes in chemical potential and temperature, showcasing positive and negative peaks in the energy range of − 2 to − 1 eV, beyond which the thermopower significantly plunges to zero. The optimum thermopower gotten at 300 K is $2050$ $\upmu \mathrm{ V }{\mathrm{K}}^{-1}$ for RbNpO_3_ and $1450$ $\upmu \mathrm{ V }{\mathrm{K}}^{-1}$ for RbPuO_3_, respectively. At higher temperatures (900 K), S is marginally reduced to $650$ $\upmu \mathrm{ V }{\mathrm{K}}^{-1}$ for RbNpO_3_ and $500$ $\upmu \mathrm{ V }{\mathrm{K}}^{-1}$ for RbPuO_3_. The computed Seebeck coefficient values have been documented in Table 3 in tandem with other published theoretical and experimental findings. It is pretty obvious that the present materials perceive higher Seebeck coefficient values than other reported perovskites due to the wider band gap and confined carrier mobility of the transport carriers. The high thermopower can also be attributed to the steep peaks in the electronic DOS rendered by the quantum confinement effect; in addition, the high effective mass of the carriers further justifies the high thermopower of the present materials. The effective mass of the carriers (holes and electrons) is furnished by the relation given as^62^; ${m}^{*}=\frac{{\mathrm{\hslash }}^{2}}{{\partial }^{2}E/{\partial }^{2}k}$. According to this equation, the effective mass of carriers is influenced by the curvature of bands, with less curved bands (flat bands) being escorted by the high effective mass and vice versa. The electronic band structure of the given materials expresses fewer curved bands in the proximity of the Fermi level, which contributes to high effective mass of the carriers and hence elevates the Seebeck coefficient since Seebeck is encountered to team up with the effective masses of the carriers, as acknowledged by relation given as^39^; $S=\frac{8{\pi }^{2}{k}_{B}^{2}}{3e{\hslash }^{2}}{m}^{*}T{\left(\frac{\pi }{3n}\right)}^{2/3}$, where the symbols correspond to their usual meanings, defined elsewhere^39^.

**Table 3:** Calculated values of Seeback coefficient (S) and Figure of merit (Z_e_T) for present study in comparison with (a) theoretical and (b) experimental results available in the literature.

(a) Theoretical comparisons							
Parameter	RbNpO_3_ [present]	RbPuO_3_ [present]	SrMnO_3_^34^	GdMnO_3_^35^	TbMnO_3_^35^	Sr_2_HoNbO_6_^36^	Sr_2_EuReO_6_^37^
Seeback coefficient (S)	2050	1450	800	264	212	− 1000	300
Figure of merit (Z_e_T)	1.01	0.987	0.979	0.8	0.88	0.97	0.98

(b) Experimental comparisons							
Parameter	RbNpO_3_ [present]	RbPuO_3_ [present]	LaCo_0.95_Ni_0.05O3_ ± $\delta$^40^	PrCo_0.95_Ni_0.05_O_3_ ± $\delta$^40^	La_0.95_Sr_0.05_CoO_3_^41^	Sr_2_RuYO_6_^42^	Sr_2_RuErO_6_^42^
Seeback coefficient (S)	2050	1450	250	340	710	480	420
Figure of merit (Z_e_T)	1.01	0.987	0.07	0.08	0.18	–	–

Furthermore, as depicted in Fig. 8a, the Seebeck coefficient declines against the rise in temperature and chemical potential. These results are exclusively in conformity with the Mott relation, which expresses the dependency of the Seebeck coefficient on chemical potential and temperature and is written as^63^; $S=\frac{{\pi }^{2}{k}_{B}^{2}T}{3e}{\left\{\frac{1dn(\varepsilon )}{n d\varepsilon }+\frac{1dn(\varepsilon )}{\mu d\varepsilon }\right\}}_{\varepsilon =\mu }$. This equation conveys that with an increase in µ and T, the Seebeck coefficient decreases due to stimulation of both types of carriers (bipolar effect). Also, the peak of thermopower is decided by the carrier concentration, as low carrier concentration facilitates high thermopower and vice versa. Thus, we can assert that both carrier concentration and temperature should be low to get maximum thermopower since high temperatures end up causing more carriers to become thermally excited, increasing mobility and letting down the Seebeck coefficient. The graphical interpretation of the Seebeck coefficient against carrier concentration presented in Fig. S2a and b (Supplementary Information file) reflects that the maximum value of thermopower is accomplished in the p-type doping zone at a minimum carrier concentration of $0.4\times {10}^{23} {\text{cm}}^{-3}.$

#### Electrical conductivity

Electrical conductivity over the relaxation time constant ($\sigma /\tau$) defines the percentage of unbound charge carriers that traverse through a material. The free carriers (electrons) capture kinetic energy as they absorb heat, relocate to cooler regions of the material, and eventually generate an electric current. To mitigate the effects of Joule's heating effect, thermoelectric materials must have a high electrical conductivity^64^. The electrical conductivity at different temperatures (300 K, 600 K, and 900 K) in rebuttal to chemical potential is addressed graphically in Fig. 8b; it can be noticeably seen that both these materials exert nearly the same electrical conductivity values. The electrical conductivity of RbNpO_3_ (RbPuO_3_) is almost non-existent in the energy range of − 1.5 eV to − 0.5 eV (− 2.0 eV to − 1.0 eV), asserting that these materials would offer decent thermoelectric output strictly beyond these specific regions. The peak values of $7.0\times$ 10^20^ (${\Omega }^{-1}{{\text{m}}}^{-1}{{\text{s}}}^{-1}$) and 7 $.12\times$ 10^20^ (${\Omega }^{-1}{{\text{m}}}^{-1}{{\text{s}}}^{-1}$) are achieved at 300 K for n-type RbNpO_3_ and RbPuO_3_, respectively; these values are incredibly good for a thermometric material and might be credited to the bulk of metallic spin electrons that make up the total electrical conductivity. Pertaining to further assessment of Fig. 8b, electrical conductivity behaves consistently at all temperatures, unlike thermopower and increases with increasing chemical potential. This is due to the fact that increasing chemical potential elevates the carrier concentration, which in turn boosts electrical conductivity, as justified by the relation; $\sigma =\frac{n{e}^{2}\tau }{m}$, where the symbols assume their typical meanings. The variations pertaining to the electrical conductivity against carrier concentration are pictured in Fig. S3a and b (supplementary information). The n-type region with a higher concentration affords the maximum value of electrical conductivity, whereas a lower carrier concentration has a contrasting effect on conductivity and practically disappears at a minimum carrier concentration of $0.4\times {10}^{23} {{\text{cm}}}^{-3}.$

#### Thermal conductivity

Heat conduction in a material is driven by two mechanisms: (a) electron or hole drift across the crystal, resulting in electronic thermal conductivity (κ_e_); and (b) phonon lattice vibrations that contribute to the lattice thermal conductivity (${\upkappa }_{l}$). The total thermal conductivity is expressed as the entirety of electronic (κ_e_) and lattice portions $({\upkappa }_{l})$ signified as; κ_t_ = κ_e_ + ${\upkappa }_{l}$, with the symbols assuming their usual meanings. In the present work, we sought to anticipate the collective behaviour of both electronic and lattice thermal conductivity, besides predicting the variation of both these entities individually. Lattice, electronic, and total thermal conductivity are all represented graphically as a function of temperature and can be traced in Fig. S5a and b (Supplementary Information File).

Firstly, we will make sense of the lattice thermal conductivity. As stated earlier, phonon lattice vibrations are directly to blame for the lattice thermal conductivity, which itself is affected by a number of variables, including phonon scattering and dispersion as well as Gruinsen and Debye factors^39,65^. The Slack model explicitly predicts the appropriate behaviour of ${\upkappa }_{l}$ at any temperature and is designated numerically as^39^: ${\upkappa }_{l}=\frac{A{\theta }_{D}^{3}{V}^{1/3}m}{{\gamma }^{2}{N}^{2/3}T}$. The Slack model emphasizes that the Debye temperature (${\theta }_{D}$), Gruneisen parameter ($\gamma$), temperature (T), volume (V), average molar mass per atom (m), and the number of atoms per unit cell (N) all impact the lattice thermal conductivity. The parameter A is determined as^39^; $A=\frac{2.43\times {10}^{8}}{1-\frac{0.514}{\gamma }+\frac{0.228}{{\gamma }^{2}}}$. Utilizing these associated factors, the Slack model has been implemented to calibrate the lattice thermal conductivity. As can be observed from Fig. S5a and b, the ${\upkappa }_{l}$ of two materials precipitously diminishes as the temperature escalates since lattice thermal conductivity is intrinsically more prominent at low temperatures and speedily declines in dissent to rising temperatures. The decreasing trend in ${\upkappa }_{l}$ with rising temperature may be characterized by excessive phonon scattering. The expected lattice thermal conductivity values are $16 \, {\text{W/mK}}$ for RbNpO_3_ and $14 \, {\text{W/mK}}$ for RbPuO_3_, respectively. Further perception of the Fig. S5a and b reveals insights into the electronic and total thermal conductivity along selected range of temperature. It is imperative to note that the behaviour of electronic thermal conductivity upsurges with temperature, concealing its pre-eminence at higher temperatures since the carrier concentration increases significantly as the temperature goes up, letting κ_e_ to trounce at higher temperatures. The total thermal conductivity at first proceeds in the same direction as ${\upkappa }_{l}$, but as the temperature is raised, the advancement of κ_e_ pushes κ_t_ to adapt its pattern. The corresponding κ_t_ values for RbNpO_3_ and RbPuO_3_ at room temperature are 4 ${\text{W/mK}}$ and 3 ${\text{W/mK}},$ respectively. Further analysis of Fig. S5a and b explicitly depicts that ${\upkappa }_{l}$ predominates below 250 K, whereas κ_e_ proliferates at all temperature ranges above 250 K. The low value of ${\upkappa }_{l}$ at high temperatures (above 250 K) applauds better TE performance since they make up a small fraction of the total thermal conductivity and thus have slight impact on the thermoelectric efficiency of these material.

In addition to temperature, the chemical potential dependency of electronic thermal conductivity has further been traced in Fig. 8c; infers the κ_e_ of each of these materials tends to rise as temperature goes up, possibly because as temperature is raised, bound electrons become flustered by acquiring thermal energy and thus become accessible for conduction. To sustain the temperature gradient, the thermal conductivity of an impactful thermoelectric device must be limited. For n-type RbNpO_3_ and RbPuO_3_, the estimated values of κ_e_ at 300 K are 4.9 $\times$ 10^15^ $({\text{Wm}}^{-1}{{\text{K}}}^{-1}{{\text{s}}}^{-1})$ and of 5.0 $\times$ 10^15^ $({\text{Wm}}^{-1}{{\text{K}}}^{-1}{{\text{s}}}^{-1})$ respectively. Such values are considerably lower and hence appeases the high thermoelectric efficiency of these materials. The κ_e_ and $\sigma$ have a striking similarity against chemical potential as they are linked through a well-known connection known as the Weidman–Franz Law^66^, stated as; κ_e_ $=L\sigma T$. The κ_e_ of these materials expands in the negative doping region, just as the electrical conductivity; nevertheless, the temperature effects seem to be more perceptible in κ_e_ than in $\sigma$, as evidenced in Fig. 8c. Furthermore, the carrier concentration plays a decisive role in uplifting the electronic thermal conductivity as depicted by the graphical plots of κ_e_ versus carrier concentration shown in Fig. S4a and b (Supplementary information). These plots consent us to extrapolate that the κ_e_ fades away at the lowest carrier concentration.$(0.4\times {10}^{23} {{\text{cm}}}^{-3})$, while it intensifies to its maximum at higher concentrations $(-2.0.\times {10}^{23} {{\text{cm}}}^{-3})$.

#### Thermoelectric efficiency

Power factor (PF) and figure of merit are practical metrics designed to quantify a material's thermoelectric performance (ZT). The power factor outlines the thermopower generating efficiency of any material and may be computed using the association between thermopower and electric conductivity given as;$\mathrm{PF}= {\mathrm{S}}^{2}\upsigma$. As per the basic equation, a high-power factor necessarily involves excellent thermopower and electrical conductivity. Figure 8d reflects the graphical variation of power factor against chemical potential at distinct Kelvin temperatures (300 K, 600 K, and 900 K). On moving from 300 to 900 K triggers the power factor for both compounds to increase. At 900 K, the peak values of 19 × 10^11^ ${\text{W/K}} \, {\text{ms}}$ for RbNpO_3_ and 11 × 10^11^ ${\text{W/K}} \, {\text{ms}}$ are gathered in the negative energy range of chemical potential. At low temperatures, the PF values for RbNpO_3_ and RbPuO_3_ diminish slightly to 7.5 × 10^11^ ${\text{W/K}} \, {\text{ms}}$ and 3 × 10^11^ ${\text{W/K}} \, {\text{ms}}$ respectively. The estimated PF values are consistently greater than those for other comparable perovskites reported in the literature^22,29,39^, most likely as a consequence of the momentous Seebeck coefficient values.

The authentic performance of a thermoelectric material is decided by the dimensionless figure of merit (zT), which takes into account the power factor and thermal effects. A material with a zT equal to or greater than one is deemed appropriate for thermoelectric applications. Among the thermal effects, just electronic thermal conductivity has been acknowledged since, as previously discussed, lattice thermal conductivity has a negligible impact at high temperatures. Also, BoltzTraP generates reliable data for electronic thermal conductivity and doesn’t encompass specifications for lattice thermal conductivity. Consequently, electronic zT has been predicted without assuming lattice thermal conductivity to facilitate effortless comparison with other published results. The illustration of electronic figure of merit (zT) as a function of chemical potential at various temperatures is graphically depicted in Fig. 8e. The maximum value of zT tracked down for RbNpO_3_ and RbPuO_3_ at 300 K is 1.01 and 0.987, respectively. Despite being subjected to higher temperatures, the zT values for the given materials do not descend to zero, as portrayed in Fig. 8e. It is evident from comparing the findings of the given materials that RbNpO_3_ bears a considerably higher zT value than RbPuO_3_ due to its incredibly increased Seebeck coefficient. The estimated values of electronic figure of merit (zT) are higher than other existing theoretical and experimental results, as summarised in Table 3. The high zT for the present compounds is facilitated by the exceedingly high PF values, which in turn are replicated by the higher Seebeck coefficient and decent electrical conductivity values. Thus, we can logically conclude that RbMO_3_ perovskites could depict amazing thermoelectric performance, both at low and high temperature ranges, and therefore can be viewed as possible nominees for competent thermoelectric device applications.

### Thermal properties

**Figure 9:**
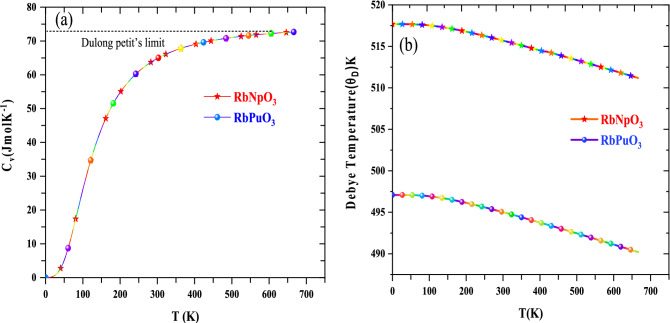
Thermodynamic features of RbMO_3_ perovskites as a function of temperature: (**a**) Specific heat, (**b**) Debye temperature.

The thermodynamic properties are very important for understanding the typical behaviour of materials under high temperature and high-pressure environments. The Gibbs2 code within the quasi-harmonic regimes has been effectively employed to scrutinize the thermodynamic behaviour of RbMO_3_ perovskites^54^. In this section, the specific heat capacity (*C*_v_), Debye temperature $({\theta }_{D})$, Gruneisen parameter $(\gamma )$, and thermal expansion $(\alpha )$ of RbMO_3_ perovskites have been analysed at zero pressures over a selected temperature range. Specific heat (*C*_v_), which is interpreted as a material's ability to retain heat at ambient temperature, outlines the distinctive behaviour of solids under diverse temperature and pressure conditions. Figure 9a depicts the variation of specific heat at constant volume (C_v_) against temperature; inferring two distinct sorts of variations. First, at low temperatures, the heat capacity (C_v_) increases dramatically between 0 and 300 K, in accordance with the Debye ${T}^{3}$ law, since only longwave phonons are energised in this temperature range^67^. Second, all frozen phonons above 600 K undergo thermal excitation, which inevitably leads to the classical Dulong Petit's Limit^68^. The predicted *C*_V_ values for both compounds are 72 ${\text{J/mol {K}}}^{-1}$, which are closely in line with previously published theoretical results^22,27^. The identical C_v_ values for both the compounds can be explained by the fact that specific heat advances the classical Dulong law which is given by; ${C}_{V}=3nR,$ where *R* is the gas constant and $n$ number of molecules ($n$ is same for both these alloys. The Dulong petit's law holds true for all monoatomic solids, hence striking the similar values of C_v_ for the allotted materials owing to the equivalent number of atoms/molecules.

The Debye temperature is a critical physical parameter in materials, defining the boundary between classical phonon activity and quantum–mechanical aspects. Above the Debye temperature, a crystal shows classical behaviour because the phonon contribution to heat capacity outweighs the quantum impact in terms of heat capacity. Figure 9b shows a plot of the variation of Debye temperature against temperature. In a certain temperature range, the Debye temperature of the two materials is consistent, which could be associated with a decline in anharmonicity at low temperatures, leading to constant Debye temperature. Nevertheless, as the temperature exceeds, the Debye temperature drops, shifting the vibration spectra of the corresponding atoms. The estimated values of Debye temperature (θ_*D*_) for RbNpO_3_ and RbPuO_3_ at 0 GPa are $510 {\text{K}} {\text{and}} 497 {\text{K}}$, respectively; these values are significantly higher than other reported comparable perovskites in view of the high bulk modulus and low molar mass of the pristine compounds^24–26^. The elevated values of Debye temperature imply the higher temperature application stand of the given materials. The discussion over Gruneisen parameter $(\gamma )$, and thermal expansion $(\alpha )$ is presented in the supplementary information file.

### Mechanical stability

The fundamental characteristics of a material like plasticity, elasticity, strength, hardness, and ductility, are unequivocally evidenced by its mechanical properties, convincing us to look into the mechanical behaviour of novel RbMO_3_ compounds. In DFT simulations, the elastic constants are frequently used to track down the mechanical response since they typically correlate a material's dynamical behaviour to its mechanical response. For the given materials, the elastic stiffness constants (Cij) within the regimes of Thomas Charpin's cubic elastic package^53^ integrated with the Wien2k code have been computed to provide a qualitative and meticulous description of their elastic stability. For cubic symmetry crystals, just three stiffness constants, *C*_11_, *C*_12_, and *C*_44_, are intended to sketch their elastic properties. The longitudinal distortion, *C*_11_, reflects the hardness while *C*_12_ signifies Poisson's proportions and *C*_44_ specifies the shear modulus; the projected values of these constants are listed in Table 4. The positive value of each constant discloses the stability of the given compounds. Additionally, the well-known Born stability criterion necessitates some restrictions on elastic constants, which are listed as^69^ as; ${C}_{11}-\left|{C}_{12}\right|>0$; ${C}_{12}<B<{C}_{11};$ ${C}_{44}>0;$ ${C}_{11}>0; {C}_{11}+2{C}_{12}>0.$ The outlined elastic constants legitimise the stability of the specified materials by fully satisfying all limiting constraints imposed by Born criteria. Further perceptions of the elastic constants emphasize the anisotropic and damage-resistance characteristics of RbMO_3_ perovskite inferable from high and low values of *C*_11_ and *C*_44_. The systematic manipulation of elastic stiffness constants renders different significant parameters (B, G, Y, *v*) that impact the elastic stability of a specific material. The bulk (B) and shear moduli (G) have been derived by using the Viogt–Reuss–Hill approximations presented in the supplement section^70–72^. In addition, the Young's modulus(Y), Poisons' ratio (*v*), Kleiman parameter (ξ), and Lame's parameters (λ, β)^73^ were projected utilising specific equations noted in the supplementary information file. All these parameters have been calculated for the present materials, and the values are listed in Table 4. It is worth to mention that bulk and shear are well-known criteria to reckon the hardness of a material and their values predicted for the present compounds are 133 (124) GPa and 59 (54) GPa for RbNpO_3_ (RbPuO_3_), respectively, both of which are pretty decent and, as such, anticipate the stiffness of these materials. Also, the high bulk value with low shear value upholds the damage tolerance, easily machinable, and rigidity of these materials. Likewise, the significant values of Young’s modulus (*Y*) and Lame’s coefficients (λ, β) tend to indicate the strength and compressibility of these materials; nevertheless, RbNpO_3_ offers superior values of Y, λ, and β than RbPuO_3_ due to its high bulk and shear values, intimating that RbNpO_3_ is slightly more compressible and stiffer than RbPuO_3_. Meanwhile, a low Kleiman parameter (ξ) value specifies the higher resistance of titled materials to bond bending and bond angle distortions since a low value Kleiman parameter (ξ) augments higher resistance against bond bending and bond angle distortions and vice-versa^73^. All the above- calculated parameters have substantial ramifications in modern engineering science, and their elevated values for the present materials allow them to be used in a wide spectrum of technological applications. Furthermore, the computed values of B, G, Y, λ, and β and the findings of other reported compounds^24–26,28,73^ are in close agreement, with some parameters preserving higher values for the given materials, specifying that they can be more feasible choices for diverse technological applications.

**Table 4:** Calculated elastic constants ${\mathrm{C}}_{11}$, ${\mathrm{C}}_{12}$, and ${\mathrm{C}}_{44}$ (GPa); bulk modulus B (GPa); shear modulus G (GPa) Young’s modulus Y (GPa); Poisson’s ratio ν; B/G ratio; Cauchy’s pressure (${\mathrm{C}}_{12}-{\mathrm{C}}_{44}$), Kleiman’s parameter ($\xi )$ and Lame’s constants $\left(\uplambda ,\beta \right)$ for RbMO_3_ compounds.

Compound	*C*_*11*_	*C*_*12*_	*C*_*44*_	*B*	*G*	Y	*v*	*B/G*	*C*_*12*_*–C*_*44*_	*ξ*	*λ*	*β*
RbNpO_3_	260.57	69.87	42.50	133.4	59.12	154.5	0.30	2.41	27.37	0.41	89.13	59.42
RbPuO_3_	235.15	68.71	40.64	124.2	54.18	141.9	0.30	2.29	28.03	0.44	81.86	54.57
BaPuO_3_	221.82^a^	72.18^a^	46.26^a^	122.0^a^	56.68^a^	150.0^a^	0.30^a^	2.17^a^	25.95^a^	–	–	–
BaNpO_3_	241.18^b^	64.58^b^	44.95^b^	128.1^b^	62.30^b^	160.9^b^	0.29^b^	2.07^b^	19.63^b^	–	–	–
										0.31^c^	48.37^c^	37.8^c^

[a]^25^, [b]^24^, [c]^73^. The results of other perovskites^24,25,73^ are tabulated for comparison.

To be further persuaded of mechanical stability, the ductility or brittleness of these perovskites must be acknowledged, which can frequently be predicted dynamically using the Cauchy pressure factor, Pugh ductility index, and Poisson's proportions^74,75^. The Cauchy's pressure, characterized as (*C*_12_-*C*_44_), manifests the ductile or malleable character relying on whether the value of Cauchy's pressure is positive or negative. Pugh's ratio empirically ties a material's plastic properties to its elastic moduli by a factor of B/G, and it sets up a critical value of 1.75 above which a material is deemed ductile and below which it is malleable. Poisson's ratio, incorporated by Frantsevich et al.^76^, captures the brittleness or ductility of a specific material. If Poisson's ratio varies as *v* > 0.26, the material is assumed to be ductile; conversely, *v* < 0.26 makes it malleable. All three parameters have been calculated for the titled alloys, and the results are summarised in Table 4. The fact that Cauchy's pressure is positive and that Pugh's index value (Poisson’s ratio) is higher than its critical value of 1.75 (0.26) predicts the ductility of these materials. These values have also been compared to those of other similar reported perovskites^24,25^ and are found to be in good agreement, justifying our findings. The ductile character of these materials commends them for devising various tools of different sizes and dimensions. Further calculations have been performed to determine the Vickers hardness $({H}_{V})$ and melting temperature (${T}_{m}(K$), two properties that are intrinsically linked to elastic constants. The melting temperature has been evaluated using the equation given as^24,25^; ${T}_{m}(K)=\left[553(K)+\left(5.911\right){C}_{11}\right] \, {\text{GPa}} \pm 300$*.* The predicted melting temperatures are $2087.32 \pm 300 K$ and $1964.32 \pm 300 K$ for RbNpO_3_ and RbPuO_3_, respectively. The calculated ${T}_{m}(K)$ values for both compounds are considerably higher than those of other reported perovskites^24–26,28^, reflecting their tendency to preserve ground state structure across a wide temperature range.

The ${H}_{V}$ (Vickers hardness) of RbMO_3_ have been anticipated using different estimates suggested by Teter et al.^77^, ${(\mathrm{H}}_{\mathrm{V}}{)}_{\mathrm{Teter}}=0.151\mathrm{ G}$, Tian et al.^78^, ${(\mathrm{H}}_{\mathrm{V}}{)}_{\mathrm{Tian}}=0.92(\mathrm{G}/\mathrm{B}{)}^{1.137}{\mathrm{G}}^{0.708}$, and Chen et al.^79^, ${(\mathrm{H}}_{\mathrm{V}}{)}_{\mathrm{Chen}}=2{\left[(\mathrm{G}/\mathrm{B}{)}^{2}\mathrm{G}\right]}^{0.585}-3$.The projected values of these parameters are enumerated in Table 5. All of the calculated H_V_ values for both alloys are positive across all approximations, unveiling their hardness. The unavailability of the scientific data bars us to compare these results; however, they can act as reference data for future works.

**Table 5:** Intended values of Vickers Hardness ${(H}_{V})$, Anisotropic parameters and extrema of Poisson’s ratio for RbMO_3_ materials.

Material	${H}_{V}$ $\left(Teter\right)$	${H}_{V}$ $(Tian$*)*	${H}_{V}$ $(Chen)$	A_Z_	A_G_	A_U_	A_L_	*v* (100,001)	*v* $\left(110, 110\right)$
RbNpO_3_	8.92	6.55	5.39	0.44	0.08	0.20	0.08	0.11	0.54
RbPuO_3_	8.18	6.05	4.82	0.48	0.06	0.60	0.25	0.13	0.53

Furthermore, the longitudinal and transverse elastic wave velocities have been estimated using the pertinent bulk and shear values, together with the density of these compounds as^24,25^; ${v}_{t}={\left[\frac{G}{\rho }\right]}^\frac{1}{2} and {v}_{l}={\left[\frac{3B+4G}{3\rho }\right]}^\frac{1}{2}.$ The predicted longitudinal (transverse) wave velocities for RbNpO_3_ and RbPuO_3_ in (*ms*^*-1*^) are 5507 (2912) and 5162 (2762), respectively. The high B and G of these compounds enables them to have greater ${v}_{t}$ and ${v}_{l}$ values than previously recorded perovskites^24–26,28^.

Since cubic structure exhibits pure longitudinal and transverse modes along distinct axes through the [1 0 0], [1 1 0], and [1 1 1] directions, which can be either transverse or quasi-longitudinal, were determined using Brugger's relation^80^; *v* = $\sqrt{\frac{Ceff }{\rho }}$. The values of *C*_eff_ in various directions and the predicted wave velocities are listed in Table S3 (supplementary information).

**Figure 10:**
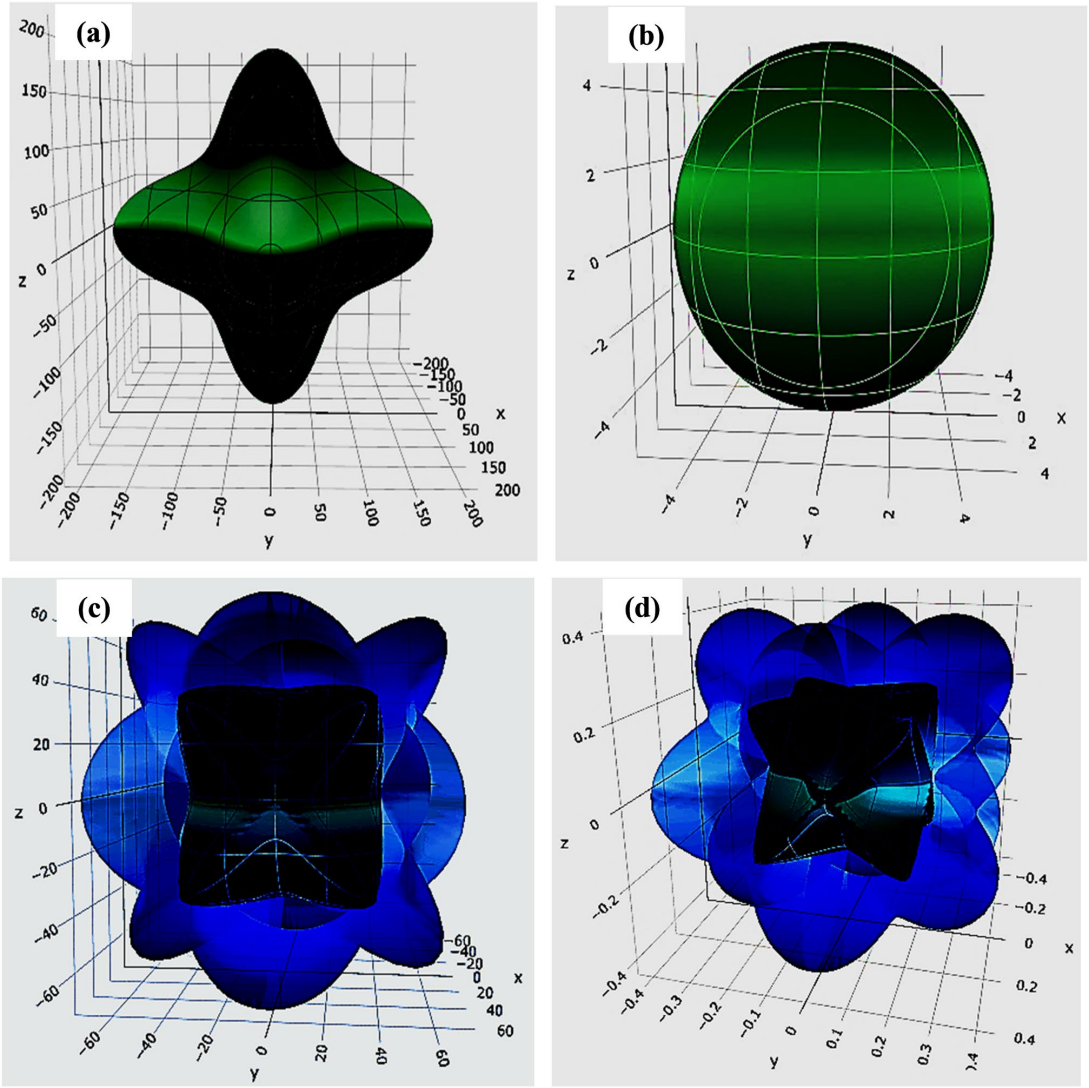
Three-dimensional graphics of (**a**) Young's modulus (*Y*), (**b**) linear compressibility (β), (**c**) shear modulus(*G*), and (**d**) Poisson's ratio(*v*) for RbNpO_3_ compound.

**Figure 11:**
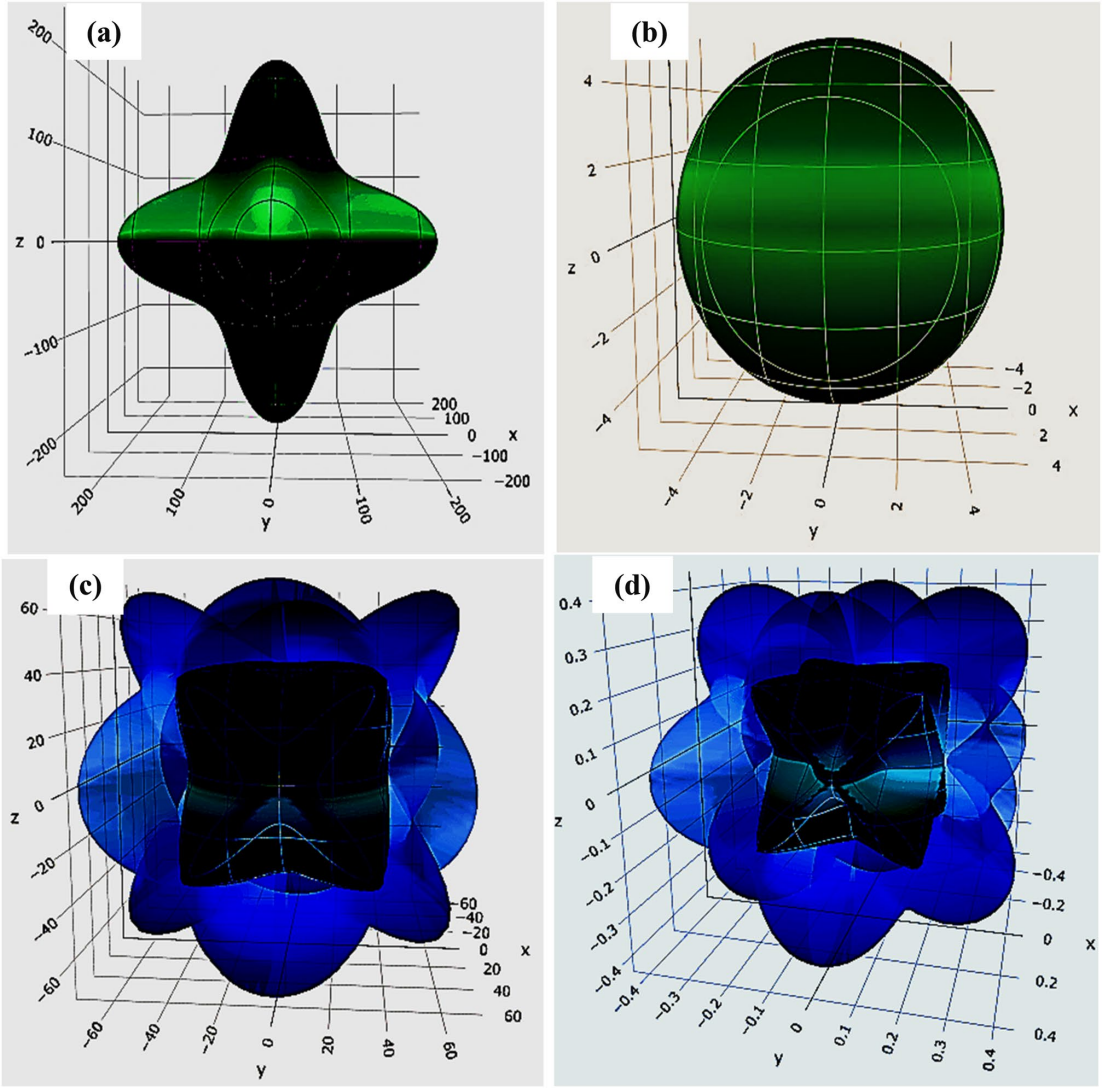
Directional dependence of (**a**) Young's modulus (*Y*), (**b**) linear compressibility(β), (**c**) shear modulus(*G*), and (**d**) Poisson's ratio(*v*) for RbPuO_3_ compound.

Mechanical anisotropy, one of the core components impacting different physical operations of a material, has been the focus of the present investigation to gain insights into the anisotropic mechanical response of RbMO_3_ compounds. The Elate Code^81^ has been used to design three-dimensional (3-D) contour plots of Young's modulus (*Y*), linear compressibility (β), shear modulus (*G*), and Poisson's ratio (*v*) to conceptualize a clear understanding of the anisotropic and elastic behaviour of the studied materials. The 3-D visual interpretations of these parameters for RbNpO_3_ and RbPuO_3_ are respectively illustrated in Figs. 10 and 11. Except for linear compressibility, all other constants diverge substantially from the entire spherical (3D) morphology, reiterating the anisotropic nature of these materials. Nevertheless, in all $(\mathrm{xy},\mathrm{ xz},\mathrm{ and} \, {\text{yz}} \, {\text{ planes}})$ directions, the same deviation from circular and spherical geometries are witnessed. Table S1 (Supplementary Information) provides an overview of the highest and lowest values of Y, B, G, and *v*. The maximum and minimum values of the *Y*-modulus in the direction [110,100] for RbNpO_3_ (RbPuO_3_) are 231.02 (204.19) and 115.26 (109.75), respectively. The *Y*_*max*_*/Y*_*min*_ ratio identifies the anisotropy in the *Y*-modulus. The shear modulus poses a star-like behaviour, expressing distinct values in various orientations, admitting the anisotropic nature of these materials. The isotropic behaviour of the bulk modulus is unveiled by its spherical shape, which is further backed by the identical values of B_V_, B_R_, and B_H_, as listed in Table S2 (Supplementary Information file). The highest and lowest values of the angular dependency for the Poisson's ratio tells the ductility of these materials as the average of the *v*_max_/*v*_min_ outcomes in value more than the index value of 0.26, as depicted in Table S1 (Supplementary Information file).

The elastic anisotropy of cubic RbMO_3_ compounds is addressed by the Zener anisotropy factor A_Z_, given as^82^;${\mathrm{A}}_{\mathrm{Z}}=1+\frac{(2{C}_{44}+{C}_{12})}{{C}_{11}}$ =$\frac{2G(1+v)}{Y}$. A_Z_ is mostly affiliated with shear anisotropy. For isotropic materials, A_Z_ has a specific value of 1. Chung and Buessem devised a new criterion of elastic anisotropy (A_G_) for cubic compounds as^83^; ${\mathrm{A}}_{\mathrm{G}}=\frac{3({A}_{Z}-1{)}^{2}}{3({A}_{Z}-1{)}^{2}+25{A}_{Z}}$. In general, the anisotropy factor (A_G_) is expressed in percentage, and a material is deemed “isotropic if A_G_ = 0. All of the components of a material's elasticity tensor are associated to the universal elastic anisotropy factor (A_U_), which is estimated as^84^; ${\mathrm{A}}_{\mathrm{U}}=5\frac{{G}_{V}}{{G}_{R}}-5$. Another anisotropy factor for cubic crystals is the Logarithmic universal Euclidean anisotropy, which is linked to the Voigt and Reuss restrictions on the bulk modulus and is expressed as^85^; ${\mathrm{A}}_{\mathrm{L}}=\sqrt{5}\mathrm{ln}\left(\frac{{G}_{V}}{{G}_{R}}\right)$. All the values allied with A_Z_, A_G_, A_U_, and A_L_ are presented in Table 5; the non-zero values of these parameters characterize the anisotropic character of the given materials. The peak values of Poisson's ratio for a cubic compound are generated by the strain along $\langle 110\rangle$ and the equivalent transverse strain along $\langle 001\rangle$ and $\langle 110\rangle$. In particular, corresponding to these directions, Poisson's ratio is specified as^86^; *v* $\left(110, 001\right)=\frac{{2A}_{Z}{C}_{12}}{3B+{A}_{Z}{C}_{11}}$ and, *v *$\left(110, 110\right)=\frac{3B-{A}_{Z}{C}_{11}}{3B+{A}_{Z}{C}_{11}}$*.* The extrema of Poisson's ratio comply with the order $\left(110, 110\right)> \left(110, 001\right)$, as depicted in Table 5, intimating that transverse strain may ensue along this specific direction for the present perovskites.

## Summary and outlook

To summarize, the present study thoroughly investigated the structural, spintronic, transport, and mechanical properties of novel *f-*electron-based RbMO_3_ perovskites. The tolerance factor, structural optimizations, and cohesive energy calculations are all consistent with ferromagnetic Pm-3m cubic stability. The electronic band structure and DOS plots explicitly state the decisive half-metallic nature of the given materials, expressing them as wide band gap semiconductor materials with better advancements in sophisticated spintronics. The half-metallic behaviour might be well elucidated by the strong hybridization of 5*f*-Np/Pu states with O-*p* states, which accounts for the arrival of the half-metallic gap. The thermoelectric study, as evidenced by the Seeback coefficient, power factor, and figure of merit, reflects the significant values of these parameters for the labelled alloys, addressing them as impactful thermoelectric materials capable of being used for electricity generation, alternative energy (production) sources, and solid-state device applications. The assessment of thermodynamic quantities, like specific heat and Debye temperature, postulates a higher temperature application spectrum of these materials. Mechanical stability has been successfully accomplished through positive elastic stiffness constants, which further interpret the ductility and anisotropy of these materials, opening up their possibilities in contemporary industrial applications. The high anisotropy of these materials has been divulged by 3-D contours of several elastic parameters and certain indispensable anisotropic values. The successful predictions of the structural and mechanical stability of the studied materials will serve as a blueprint for their experimental synthesis. In addition, our study will stimulate additional research in the experimental and theoretical realms to explore possible thermoelectric and solid-state device applications of these perovskites.

## Supplementary Information

Supplementary Information.

## Data Availability

The datasets generated by the computation and thereafter analysed would be available from the corresponding author on reasonable request.
