# Supplementary material for: Scrutinized the inherent spin half-metallicity and thermoelectric response of f-electron-based RbMO3 (M = Np, Pu) perovskites: a computational assessment
Source: Sci Rep. 2022 Nov 14;12:19476. doi: 10.1038/s41598-022-22633-y (PMC9663571; doi:10.1038/s41598-022-22633-y)
Supplement: Supplementary file 1 — Supplementary Information. [file 41598_2022_22633_MOESM1_ESM.docx]

**Supplementary Information**

**Scrutinized the inherent spin half-metallicity and thermoelectric response of *f*-electron based RbMO_3_ (M= Np, Pu) perovskites: A Computational Assessment**

^*^Mudasir Younis Sofi, ^1^Dinesh C. Gupta

^*^Department of Physics, Jamia Millia Islamia, New Delhi-110025

^1^Condensed Matter Theory Group, School of Studies in Physics,

Jiwaji University Gwalior, 474011 (INDIA).

***Email: sofimudasir244@gmail.com**

**Method of calculation**

The FP-LAPW approach splits the crystal space into non-overlapping muffin tin spheres with $R_{MT}$ as the radius of MT sphere and regions beyond the muffin tin spheres known as interstitial regions that are subjected to a plane-wave basis set for eigenvalue convergence. The present simulations assume muffin tin radii of 2.50 a. u for Rb atom, 2.15 a. u (2.18) for Np (Pu) atoms and 1.75 a. u for O atoms, respectively. For non-overlapping atomic spheres, the linearized augmented plane-wave basis set with $l_{max} = 10$ and $R_{MT}K_{max}= 7$ ($K_{max}$ denotes the highest possible *k*-value and $R_{MT}$ is the radius of the smallest sphere) has been engaged to ensure charge and energy convergence. The threshold energy of - 6.0 Ry is picked as the cut-off for separating the core and valence states. The dissolution of the Brillouin zone (BZ) into a thick *k*-mesh of 2000 integration points is indispensable for the convergence of the results. Iterations for addressing the Kohn-Sham equation are permitted until the charge for consecutive cycles iterates up to 0.0001e order, and the energy for successive cycles converges up to 0.001eV.

In GGA formulism, the exchange-correlation (𝐸_𝑥𝑐_) is viewed as a derivative of the local charge density and the associated gradient. However, GGA-PBE is incompetent in comprehending the electronic structure of highly correlated *d/f* electron systems owing to the significant depreciation of the band gap typically conceivable from electron self-interactions and a lack of potential for highly localized states. Accordingly, the GGA method needs to be complemented with other modified approximations to express the properties of such systems more precisely. To achieve this, a more sophisticated potential known as TB-mBJ has been engaged in the present work [1]. The mBJ method has been adopted to envisage the precise treatment of highly correlated d/f electron systems as it is reasonably effortless and ab-initio in nature [2]. The following equations have been used to obtain the thermoelectric coefficients of the present alloys, including Seebeck (S), electrical conductivity (σ) and electronic thermal conductivity (κ_e_) [3];


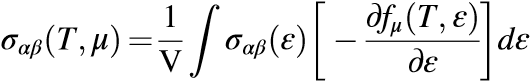
 (1)

$S_{\alpha\beta}\left( T,\mu\right)=\frac{1}{eT\sigma_{\alpha\beta}\left( T,\mu\right)}\int\sigma_{\alpha\beta}(\varepsilon)(\varepsilon-\mu)\left[ -\frac{\partial f_{\mu}(T,\varepsilon,\mu)}{\partial\varepsilon} \right]d\varepsilon$ (2)

$k_{\alpha\beta}^{0}\left( T,\mu\right)=\frac{1}{e^{2}TV}\int\sigma_{\alpha\beta}(\varepsilon)(\varepsilon-\mu)^{2}\left[ -\frac{\partial f_{\mu}\left( T,\varepsilon,\mu\right)}{\partial\varepsilon} \right]d\varepsilon$ (3)

where T, μ, V, ε, e, and f stand for temperature, chemical potential, unit cell volume, energy eigenvalue, electronic charge, and Fermi distribution function, respectively. The electrical conductivity (σ), the Seebeck coefficient (S), and the thermal conductivity (κ_e_) are the quantities represented in equations (1)– (3). Convergence of transport computations is accomplished by employing a denser *k*-mesh (100000 *k*-points).).

**Geometric structure, Volume optimization and Thermodynamic stability**


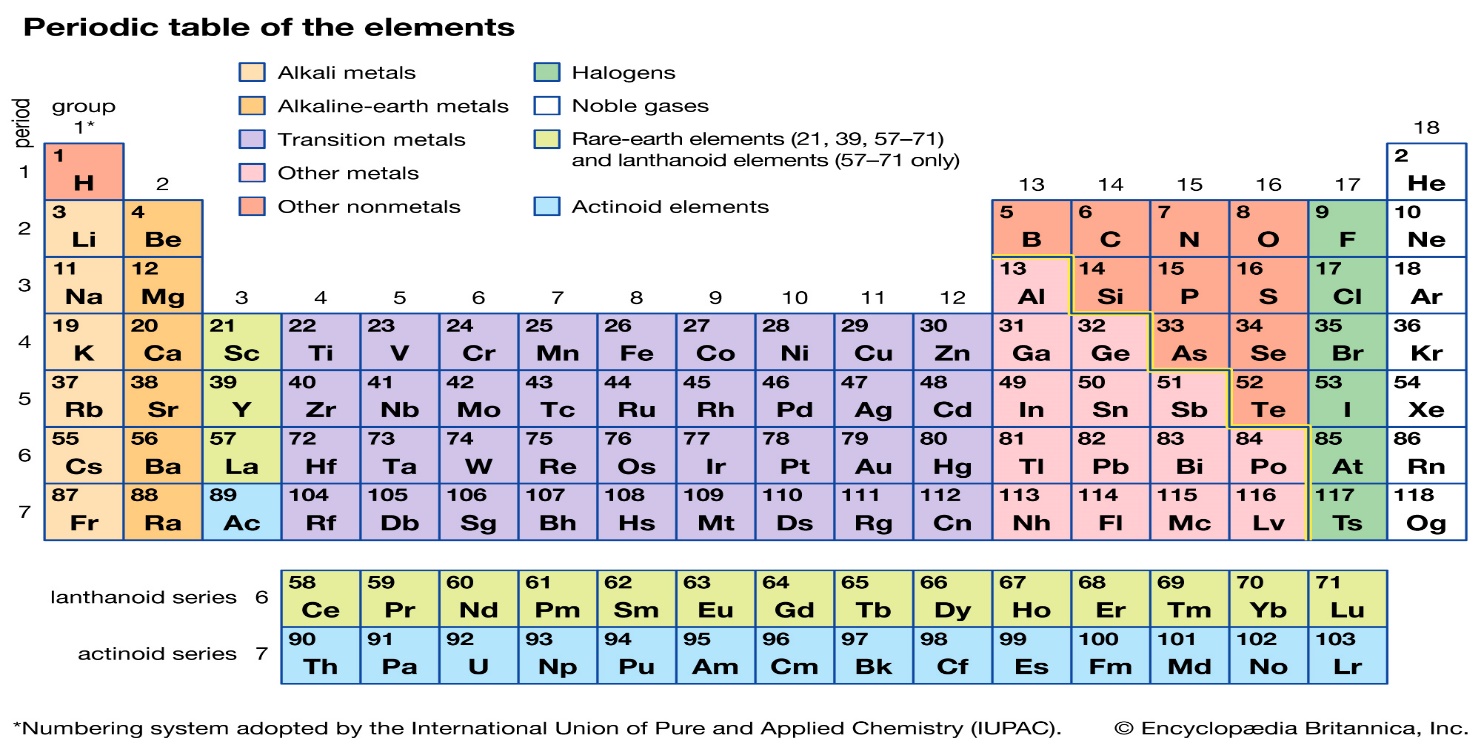


The combinations of S/p block elements with rare-earth elements constitute a broad perovskite family of ABO_3_ type compounds. In our case, Rb belongs to the s-block series containing one valence electron, M (Neptunium, Plutonium) goes to the rare-earth actinoid series containing seven/eight valence electrons, and Oxygen corresponds to the P-block series, which has six vacant p-orbital states (for three molecules). Hence, to fill its unoccupied *p*-orbital, the oxygen atom accepts one electron from the Rb atom and five electrons each from the Np/Pu atoms, leading to the formation of RbMO_3_ Perovskites.

**Rb^+1^**

**RbMO_3_**

**(Np/Pu)^+5^**

**Cohesive and formation energy**

The cohesive energy, often intimated as crystal binding energy, is an appraisal of a substance's intermolecular energy that can be approximated using the relation [4]; $E_{coh}^{{RbMO}_{3}}$=$\frac{\left[ E_{Rb}+E_{M}+3E_{O} \right]-E_{Optimized}}{5} .$Here, $E_{coh}^{{RbMO}_{3}}$ represents the cohesive energy per atom of a material. The cohesive energies per atom of the present compounds, 4.72 eV/atom for RbNpO_3_ and 4.56 eV/atom for RbPuO_3_, are decently positive, subsequently presaging that the atoms are robustly held within the lattice structures of these oxides.

The formation energy is a significant parameter that impacts the stability of a solid. The energy designed to break the links among distinctive atoms in a crystal is known as formation energy, and has been calculated for the given materials using the equation[4]; $\Delta H=E_{form}^{RbMO_{3}}= E_{total}^{RbMO_{3}}- (E$_Rb_$+ E$_M_$+ 3E$_O_), where $E_{form}^{RbMO_{3}}$ conveys the optimum energy of the system; and $E$_Rb_, $E_{M}$_(=Np, Pu)_, $E$_O_ reflects the total energy of Rubidium, Neptunium (Plutonium), and oxygen atoms in their stable elementary crystal structure. The $\Delta H$ obtained for RbNpO_3_ and RbPuO_3_ are -2.38 (eV) and -2.19 (eV), respectively.

**Critical radius**

The activation energy of oxygen migration and, in turn, the ionic conductivity in a crystal are significant factors influenced by the critical radius. The critical radius of the titled alloys was obtained using the equation [5-7]: *r_c_*=$\frac{a_{0} \left( \frac{3}{4}a_{0}-\sqrt{2r-r_{B}} \right)r_{B}+r_{B}^{2}-r_{A}}{\left( 2r_{A}-r_{B} \right)+\surd2a_{0}}$ , here *a_0_* is the optimized lattice constant, *r_A_* and *r_B_* are the ionic radii of Rb and M (M=Np, Pu) atoms, respectively. The critical radius for cubic perovskites often has a numerical value of less than 1.05. The positive value of the critical radius facilitates lower activation energy and minimum lattice disruptions. The critical radius of RbNpO_3_ and RbPuO_3_ lattices has been predicted to be 0.70 and 0.75, respectively, which are obviously much less than the critical value of 1.05. Consequently, both of our alloys have decent migration energy.

**Transport coefficients**

***Figure S1(a, b);*** *Plot of the charge carrier density (n) per unit cell against chemical potential for (a) RbNpO_3_ and RbPuO_3_ at differ rent temperatures.*

The variation of carrier concentration (n) against chemical potential in the energy range of (−2 to 2) eV is shown in Figures S1(a, b). As can be seen, carrier concentration (n) climbs as chemical potential increases and attains a minimum value in the negative region of chemical potential. The negative sign of carrier density (n) reflects electrons as the majority, whereas the positive sign depicts holes as the majority. The present compounds exhibit a negative carrier density value, indicating that the bulk of carriers are electrons and thereby suggesting the n-type behaviour of both compounds.

***Figure S2(a, b);*** *Variation of seeback coefficient with carrier concentration for (a) RbNpO_3_ and (b) RbPuO_3_ at different temperatures.*

***Figure S3 (a, b);*** *Fluctuations in electrical conductivity against carrier concentration for (a) RbNpO_3_ and (b) RbPuO_3_ at various temperatures.*

***Figure S4 (a, b);*** *Variation of electronic thermal conductivity as a function of carrier concentration for (a) RbNpO_3_ and (b) RbPuO_3_ at different temperatures.*

**Figure S5 (a, b)** Temperature-induced variations in lattice, electronic, and total thermal conductivity for (a) RbNpO_3_ and (b) RbPuO_3_.

**Thermal Properties**

**Gruinsen parameter**

A crucial thermodynamic concept known as the Gruinsen parameter (𝛾) describes the change in vibrational frequency of a lattice in response to pressure and temperature conditions. The link between the elastic and thermal behaviour of a crystal is also studied using this parameter. As reflected from the equation;$\gamma=-\frac{d\ln\theta_{D}\left( V \right)}{d\ln V}$, the Gruinsen parameter is intimately related to several other physical variables, such as the volume dependence of the Debye temperature, the bulk modulus, the calculation of the logarithmic pressure derivative of the diffusion coefficient, etc.Figure S5 (a) shows the fluctuation of $\gamma$ for RbMO_3_ oxides. When analysing the variation of $\gamma$, we see that both perovskites follow a similar increasing trend. The value of $\gamma$ parameter upsurges as the temperature rises because with increasing temperature atomic vibrations get more intense, thereby increasing anharmonicity which eventually leads to an increase in $\gamma$ . For RbNpO_3_ and RbPuO_3_, the calculated values of at O GPa and 300 K are 2.06 and 2.11, respectively.

**Thermal coefficient (**$\boldsymbol{\alpha)}$

The coefficient of thermal expansion ($\alpha)$ illustrates how temperature variations fluctuate the dimensions of an object. It is quite helpful in persuading their mechanical applications since it explicitly evaluates the fractional change in size per degree temperature at a steady pressure. The thermal coefficient is interrelated to other thermodynamic coefficients as; $\alpha=\frac{\gamma C_{V}}{B_{T}V}$, which envisages that thermal coefficient varies inversely with B_T_ but is exactly proportional to the Gruinsen parameter and Specific heat C_v_. Due to the limited reliance of B_T_ and 𝛾 on temperature, $\alpha$ exhibits the same pattern as C_v_. Figure S5 (b) illustrates the fluctuation of $\alpha$ against temperature for the present materials. The estimated values of $\alpha$ for RbNpO_3_ and RbPuO_3_ at 0 GPa and 300 K are $1.2 \times{10}^{-5}/K and 1.4\times{10}^{-5}/K$, respectively.

***Figure S5 (a, b);*** *Thermodynamic features of (a)RbNpO_3_ and (b) RbPuO_3_ against temperature.*

**Mechanical stability**

In Viogt -Reuss-Hill method, Bulk and Shear moduli are presented as;

$B_{V}=\frac{{(C}_{11}+2C_{12})}{3}$ and$G_{V} = \frac{{(C}_{11}+ C_{12}+{3 C}_{44})}{5}$ , where $B_{V}$ and $G_{V}$ are Viogt and Reuss bounds. The Bulk and Shear moduli in Reuss approximations are framed as;

$B_{V}=B_{R}$ and $G_{R}=\frac{5\left( C_{11}-C_{12} \right)C_{44}}{4C_{44}+3\left( C_{11}-C_{12} \right)}$

In the Hill approximation, the bulk and shear moduli are computed by taking the arithmetic mean of B_v_, B_R_, and G_v_, G_R_, respectively, as expressed by the following formulae;

$B= \frac{(B_{V}+R_{R})}{2}$ and $G = \frac{(G_{V}+G_{R})}{2}$

The Young's modulus and Poison's ratio are determined from the bulk and shear moduli using the relations given as;

$Y=\frac{9BG}{3GB+B}$ and *v*$=\frac{3B-Y}{6B}$

Lame’s coefficients and Kleiman parameter are given by the following equations;

$\lambda=\frac{vY}{(1+v)(1+2v)}$ , $\beta=\frac{E}{2(1+v)}$ and $\zeta=\frac{C_{11}+8C_{12}}{7C_{11}+2C_{12}}$

***Table S1*** *presents the optimum and minimal values of the Young's modulus, linear compressibility, shear modulus, and Poisson's ratio for RbMO_3_ perovskites*

| **Material** | **Young’s modulus** | | **Linear compressibility** | | **Shear modulus** | | **Poisson’s ratio** | |
| --- | --- | --- | --- | --- | --- | --- | --- | --- |
| **RbNpO_3_** | **Y _min_** | **Y _max_** | **β _min_** | **β _max_** | **B _min_** | **B _max_** | **ν _min_** | **ν _max_** |
|  | 115.26 | 231.02 | 2.4981 | 2.4981 | 42.05 | 95.35 | 0.122 | 0.550 |
| **RbPuO_3_** | 109.95 | 204.19 | 2.671 | 2.690 | 40.64 | 83.21 | 0.139 | 0.527 |

***Table S2;*** *Mean values of different elastic moduli as calculated using the Viogt-Reuss-Hill scheme.*

| **Material** | **B** | | | **G** | | | **Y** | | | **ν** | | |
| --- | --- | --- | --- | --- | --- | --- | --- | --- | --- | --- | --- | --- |
| **RbNpO_3_** | **B_V_** | **B_R_** | **B_H_** | **G_V_** | **G_R_** | **G_H_** | **Y_V_** | **Y_R_** | **Y_H_** | **V_V_** | **V_R_** | **V_H_** |
|  | 133.44 | 133.44 | 133.44 | 63.64 | 54.604 | 59.123 | 164.73 | 144.16 | 154.54 | 0.29 | 0.31 | 0.30 |
| **RbPuO_3_** | 124.45 | 124.45 | 124.45 | 57.69 | 51.06 | 54.33 | 149.79 | 134.77 | 142.29 | 0.27 | 0.30 | 0.29 |

***Table S3;*** *Calculated elastic wave velocities for RbMO_3_ perovskites along different directions.*

| **Propagation**  **Direction** | **Mode of Vibration** | ***C_eff_*** | **RbNpO_3_** | **RbPuO_3_** |
| --- | --- | --- | --- | --- |
| **[100]** | *v_l_* | *C_11_* | 6101.17 | 5754.97 |
|  | *v_t1_* | *C_44_* | 2464.02 | 2392.47 |
|  | *v_t2_* | *C_44_* | 2464.02 | 2392.47 |
| **[110]** | *v_l_* | *(C_11_+C_12_+2 C_44_)/2* | 5447.41 | 5207.94 |
|  | *v_t1_* | *C_44_* | 2464.02 | 2392.47 |
|  | *v_t2_* | *(C_11_- C_12_)/2* | 3690.72 | 3423.61 |
| **[111]** | *v_l_* | *(C_11_+2C_12_+4 C_44_)/3* | 5211.29 | 5012.33 |
|  | *v_t1_* | *(C_11_- C_12_+ C_44_)/3* | 3332.38 | 3117.85 |
|  | *v_t2_* | *(C_11_- C_12_+ C_44_)/3* | 3332.38 | 3117.85 |

**References;**

1. Tran, F., Blaha, P.: Phys. Rev. Lett. 102, 226401 (2009).
2. F. Tran, P. Blaha, K. Schwarz, Band gap calculations with Becke–Johnson exchange potential, J. Phys. Condens. Matter 19 (19) (2007) 196208.
3. I. Bourachid, M. Caid, O. Cheref, D. Rached, H. Heireche, B. Abidri, H. Rached, N. Benkhettou;

Insight into the structural, electronic, mechanical and optical properties of inorganic lead bromide

perovskite APbBr_3_ (A = Li, Na, K, Rb, and Cs), Computational Cond. Matter (2020), 24.

1. Scheidemantel, T. J., Ambrosch-Draxl, C., Tonhauser, T., Badding, J. V. & Sofo, J. O. Transport coefficients from first-principles calculations. *Phys. Rev. B* 68(12), 125210 (2003).
2. Richter J, Holtappels P, Graule T, Nakamura T and Gauckler L J 2009 *Monatsh. Chem.* 140 985.
3. Xu N, Zhao H, Zhou X, Wei W, Lu X, Ding W and Li F 2010 *J. Hydrogen Energy* 35 7295.
4. Khandy S A and Gupta D C 2017 *J. Elec. Mater*. 46 5531.
